# Supplementary material for: The Mosquito Larvicidal Activity of Lignans from Branches of Cinnamomum camphora chvar. Borneol
Source: Molecules. 2023 Apr 27;28(9):3769. doi: 10.3390/molecules28093769 (PMC10179941; doi:10.3390/molecules28093769)
Supplement: Supplementary file 1 [file molecules-28-03769-s001.zip › molecules-2328105-supplementary.pdf]

The Mosquito Larvicidal Activity of Lignans from Branches of *Cinnamomum camphora* chvar. Borneol

Zhiyong Xu [1,†], Junhui Chen [2,†], Ruifeng Shang [3], Fan Yang [4], Chuanqi Xie [1], Yunfei Liu [1], Xuefang Wen [1], Jianping Fu [1], Wei Xiong [1,*] and Lei Wu [1,*]

[1] Institute of Applied Chemistry, Jiangxi Academy of Sciences, Nanchang 330096, China; xuzhiyong@jxas.ac.cn (Z.X.)
[2] Institute of Microbiology, Jiangxi Academy of Sciences, Nanchang 330096, China; allenchen0426@gmail.com (J.C.)
[3] School of Pharmaceutical Sciences, Jiangxi University of Chinese Medicine, Nanchang 330096, China
[4] College of Food Sciences and Engineering, Jiangxi Agricultural University, Nanchang 330096, China
* Correspondence: xiongwei@jxas.ac.cn (W.X.); wulei@jxas.ac.cn (L.W.); Tel.: +86-0791-8813-1372 (L.W.)
† These authors contributed equally to this work.

Abstract: The chemical investigation of branches of *Cinnamomum camphora* chvar. Borneol guided by mosquito larvicidal activity led to the isolation of fourteen known lignans (**1**–**14**). Their structures were elucidated unambiguously based on comprehensive spectroscopic analysis and comparison with the literature data. This is the first report of these compounds being isolated from branches of *Cinnamomum camphora* chvar. Borneol. Compounds **3**–**5** and **8**–**14** were isolated from this plant for the first time. All compounds isolated were subjected to anti-inflammatory, mosquito larvicidal activity and cytotoxic activity evaluation. Compounds (**1**–**14**) showed significant mosquito larvicidal activity against *Culex pipiens quinquefasciatus* with lethal mortality in 50% (LC_{50}), with values ranging from 0.009 to 0.24 μg/mL. Among them, furofuran lignans(**1**–**8**) exhibited potent mosquito larvicidal activity against *Cx. p. quinquefasciatus*, with LC_{50} values of 0.009–0.021 μg/mL. From the perspective of a structure–activity relationship, compounds with a dioxolane group showed high mosquito larvicidal activity and have potential to be developed into a mosquitocide.

Keywords: *Cinnamomum camphora* chvar. Borneol; lignans; mosquito control; structure–activity relationship

Figure S1. ^{1}H NMR (400 MHz, $CDCl_3$) spectrum of **1**---4
Figure S2. ^{13}C NMR (100 MHz, $CDCl_3$) spectrum of **1**---4
Figure S3. ESI-MS spectrum of **1**---5
Figure S4. ^{1}H NMR (400 MHz, $CDCl_3$) spectrum of **2**---5
Figure S5. ^{13}C NMR (100 MHz, $CDCl_3$) spectrum of **2**---6

Figure S6. ESI-MS spectrum of **2**---6
Figure S7. ^{1}H NMR (400 MHz, $CDCl_3$) spectrum of **3**--7
Figure S8. ^{13}C NMR (100 MHz, $CDCl_3$) spectrum of **3**---7
Figure S9. HRESI-MS spectrum of **3**---8
Figure S10. ^{1}H NMR (400 MHz, DMSO-d_6) spectrum of **4**---8
Figure S11. ^{13}C NMR (100 MHz, DMSO-d_6) spectrum of **4**--9
Figure S12. ESI-MS spectrum of **4**---9
Figure S13. ^{1}H NMR (400 MHz, DMSO-d_6) spectrum of **5**--10
Figure S14. ^{13}C NMR (100 MHz, DMSO-d_6) spectrum of **5**---10
Figure S15. ESI-MS spectrum of **5**--11
Figure S16. ^{1}H NMR (400 MHz, DMSO-d_6) spectrum of **6**--11
Figure S17. ^{13}C NMR (100 MHz, DMSO-d_6) spectrum of **6**---12
Figure S18. HRESI-MS spectrum of **6**---12
Figure S19. ^{1}H NMR (400 MHz, DMSO-d_6) spectrum of **7**--13
Figure S20. ^{13}C NMR (100 MHz, DMSO-d_6) spectrum of **7**---13
Figure S21. ESI-MS spectrum of **7**--14
Figure S22. ^{1}H NMR (400 MHz, DMSO-d_6) spectrum of **8**--14
Figure S23. ^{13}C NMR (100 MHz, DMSO-d_6) spectrum of **8**---15
Figure S24. HRESI-MS spectrum of **8**---15
Figure S25. ^{1}H NMR (400 MHz, DMSO-d_6) spectrum of **9**--16
Figure S26. ^{13}C NMR (100 MHz, DMSO-d_6) spectrum of **9**---16
Figure S27. HRESI-MS spectrum of **9**---17
Figure S28. ^{1}H NMR (400 MHz, DMSO-d_6) spectrum of **10**--17
Figure S29. ^{13}C NMR (100 MHz, DMSO-d_6) spectrum of **10**---------------------------------------18
Figure S30. HRESI-MS spectrum of **10**--18
Figure S31. ^{1}H NMR (400 MHz, DMSO-d_6) spectrum of **11**--19
Figure S32. ^{13}C NMR (100 MHz, DMSO-d_6) spectrum of **11**---------------------------------------19
Figure S33. HRESI-MS spectrum of **11**--20
Figure S34. ^{1}H NMR (400 MHz, DMSO-d_6) spectrum of **12**--20
Figure S35. ^{13}C NMR (100 MHz, DMSO-d_6) spectrum of **12**---------------------------------------21
Figure S36. HRESI-MS spectrum of **12**--21
Figure S37. ^{1}H NMR (400 MHz, DMSO-d_6) spectrum of **13**--22
Figure S38. ^{13}C NMR (100 MHz, DMSO-d_6) spectrum of **13**---------------------------------------22
Figure S39. HRESI-MS spectrum of **13**--23
Figure S40. ^{1}H NMR (400 MHz, DMSO-d_6) spectrum of **14**--23
Figure S41. ^{13}C NMR (100 MHz, DMSO-d_6) spectrum of **14**---------------------------------------24
Figure S42. HRESI-MS spectrum of **14**--24
Table S1-14. Comparison of ^{1}H and ^{13}C data between compounds **1-14** and those in literature--25

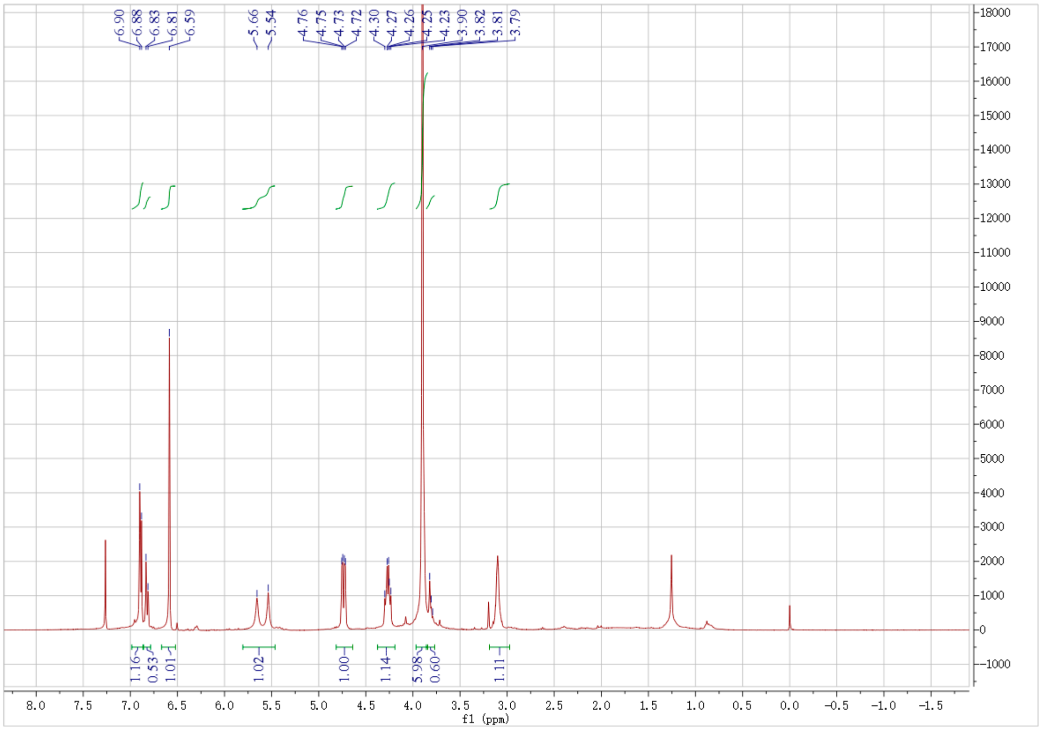

Figure S1. ^{1}H NMR (400 MHz, $CDCl_3$) spectrum of **1**

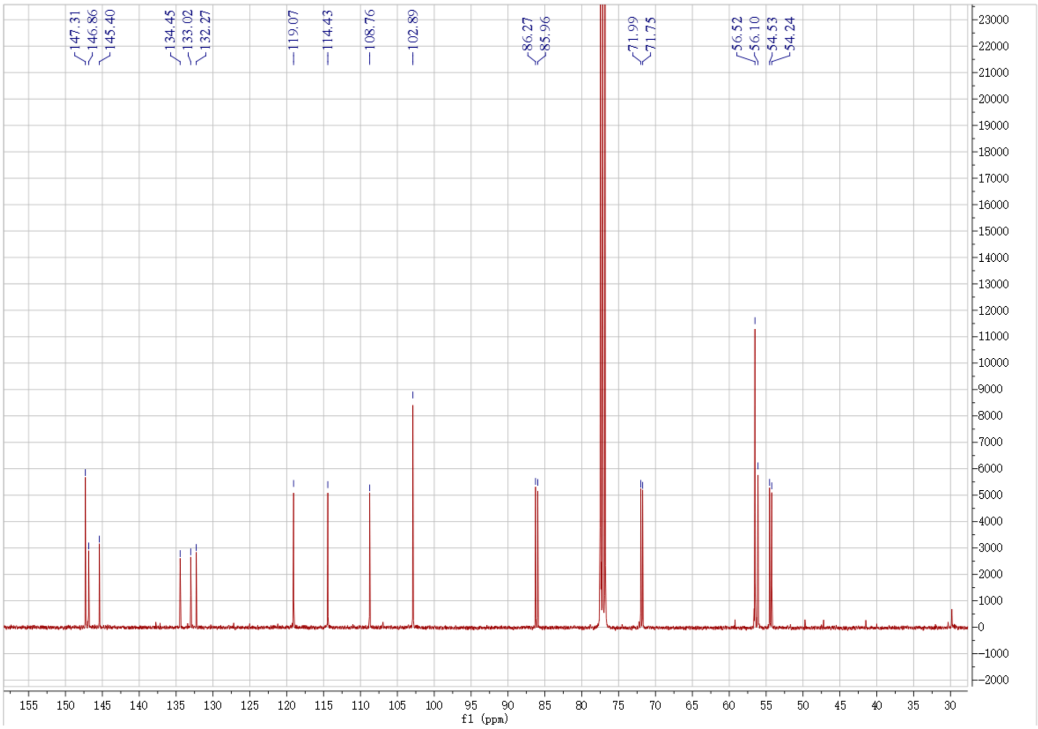

Figure S2. ^{13}C NMR (100 MHz, $CDCl_3$) spectrum of **1**

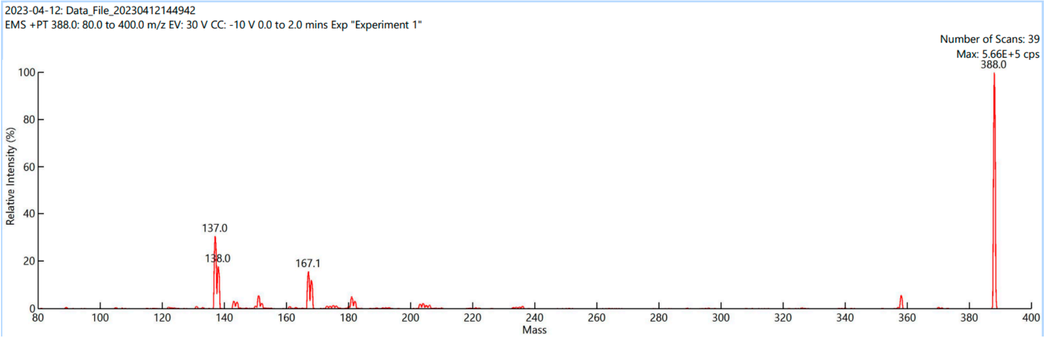

Figure S3. ESI-MS spectrum of **1**

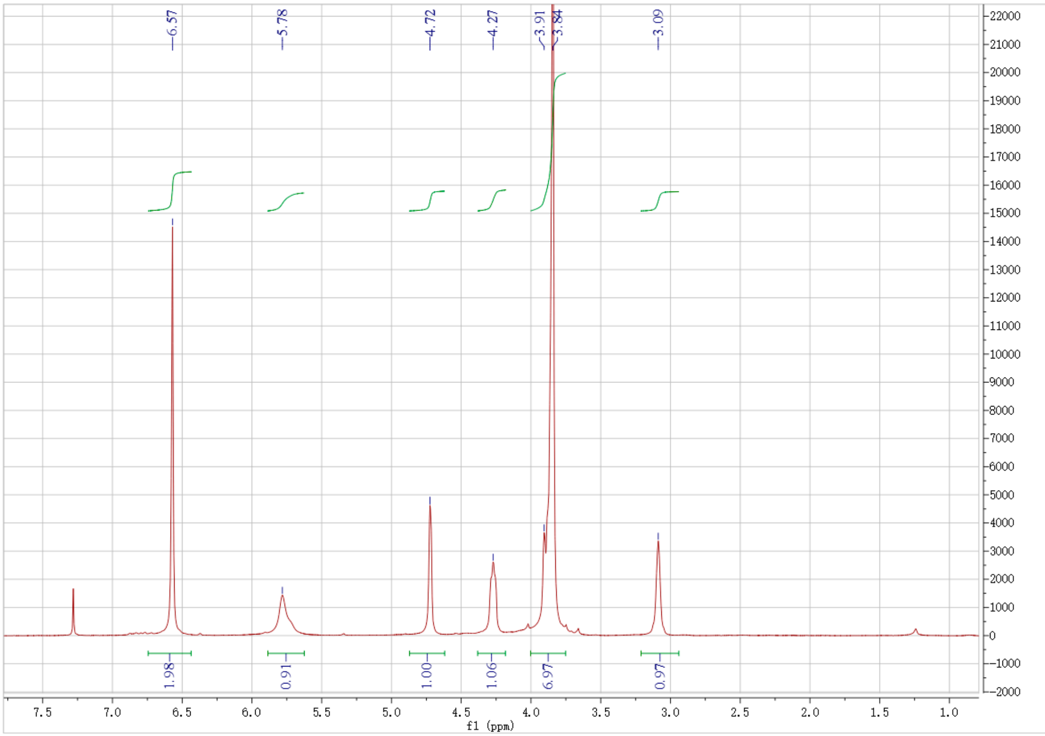

Figure S4. ^{1}H NMR (400 MHz, $CDCl_3$) spectrum of **2**

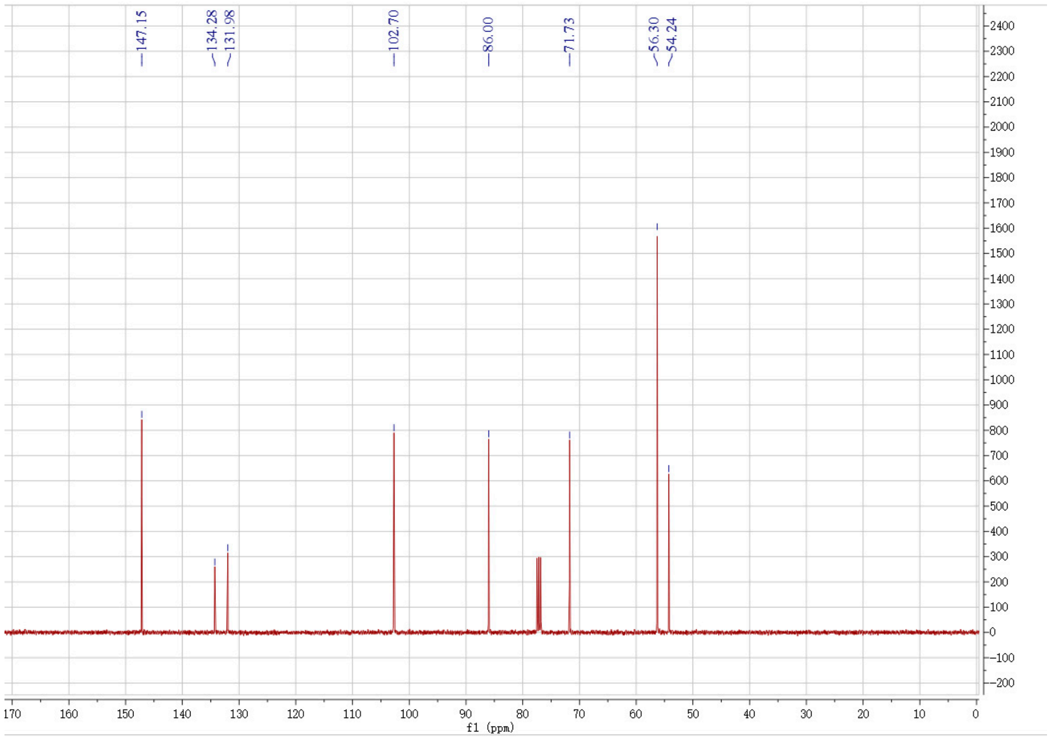

Figure S5. ^{13}C NMR (100 MHz, $CDCl_3$) spectrum of **2**

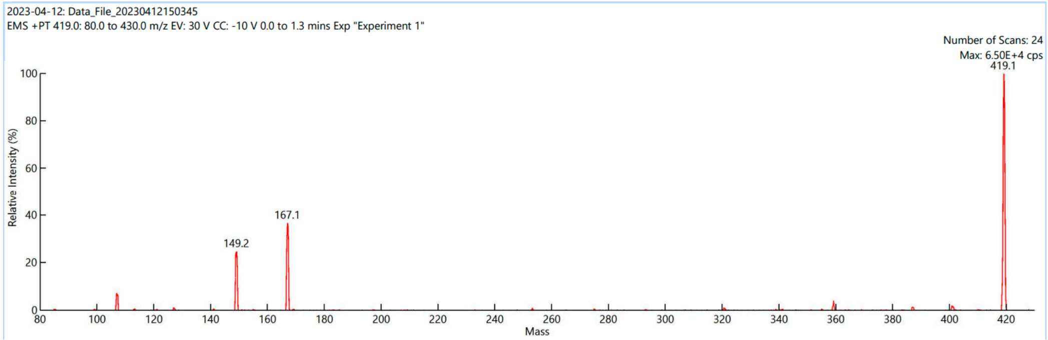

Figure S6. ESI-MS spectrum of **2**

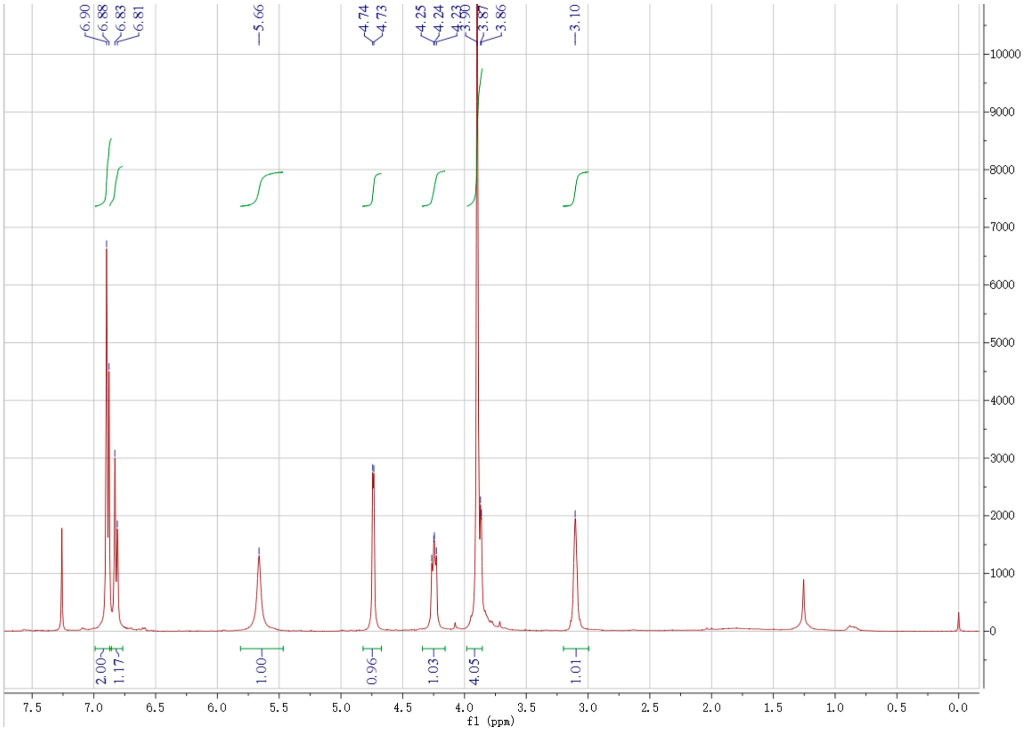

Figure S7. ^{1}H NMR (400 MHz, $CDCl_3$) spectrum of **3**

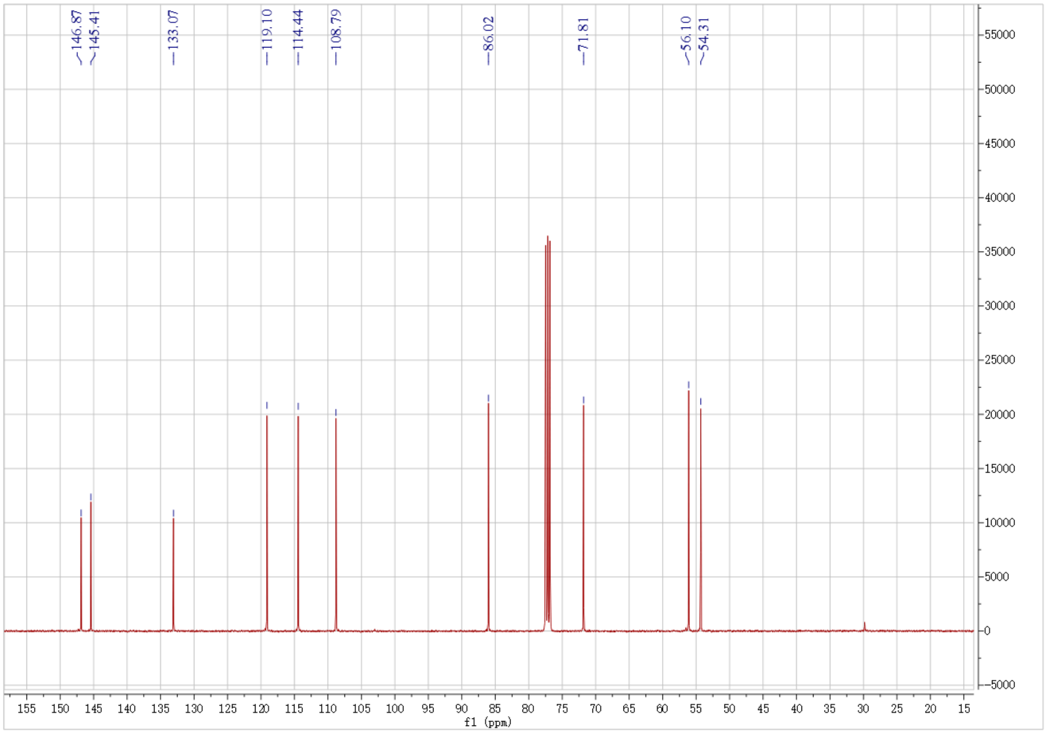

Figure S8. ^{13}C NMR (100 MHz, $CDCl_3$) spectrum of **3**

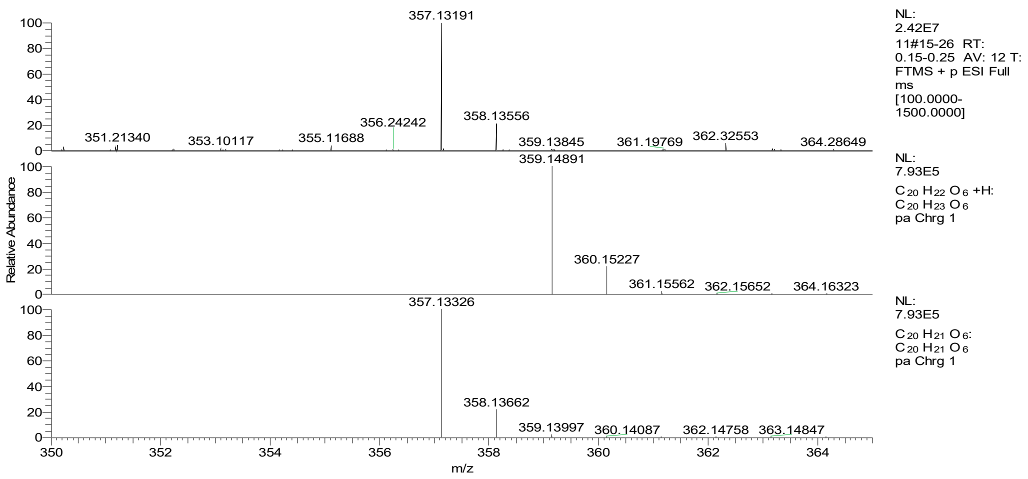

Figure S9. HRESI-MS spectrum of **3**

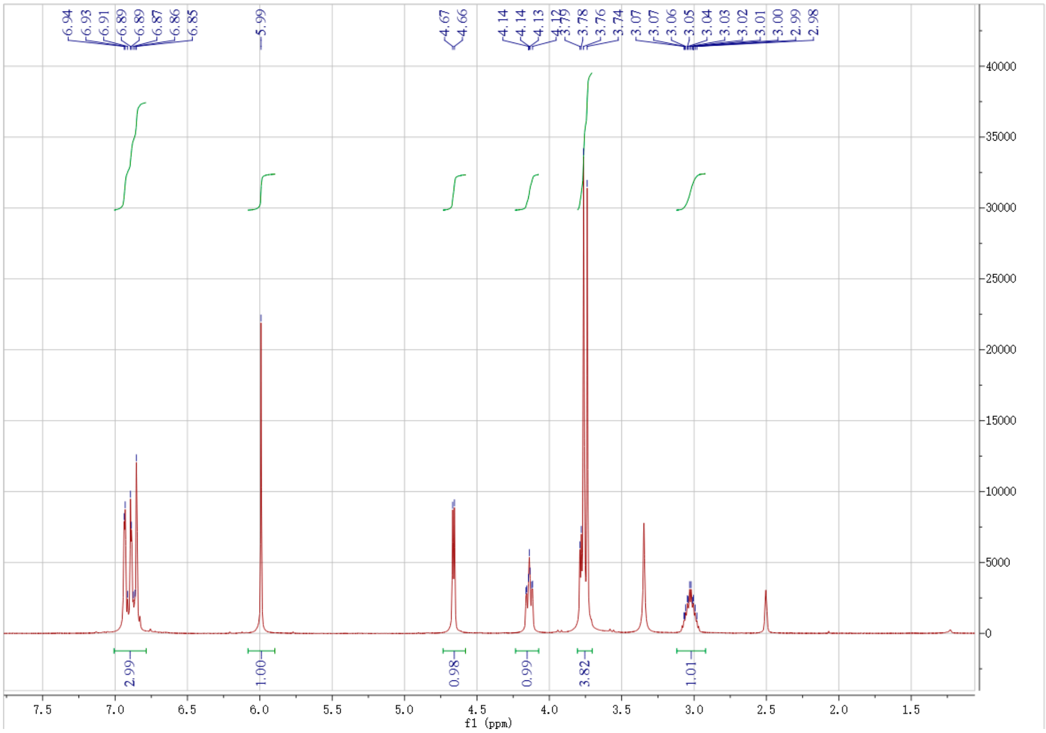

Figure S10. ^{1}H NMR (400 MHz, DMSO-d_6) spectrum of **4**

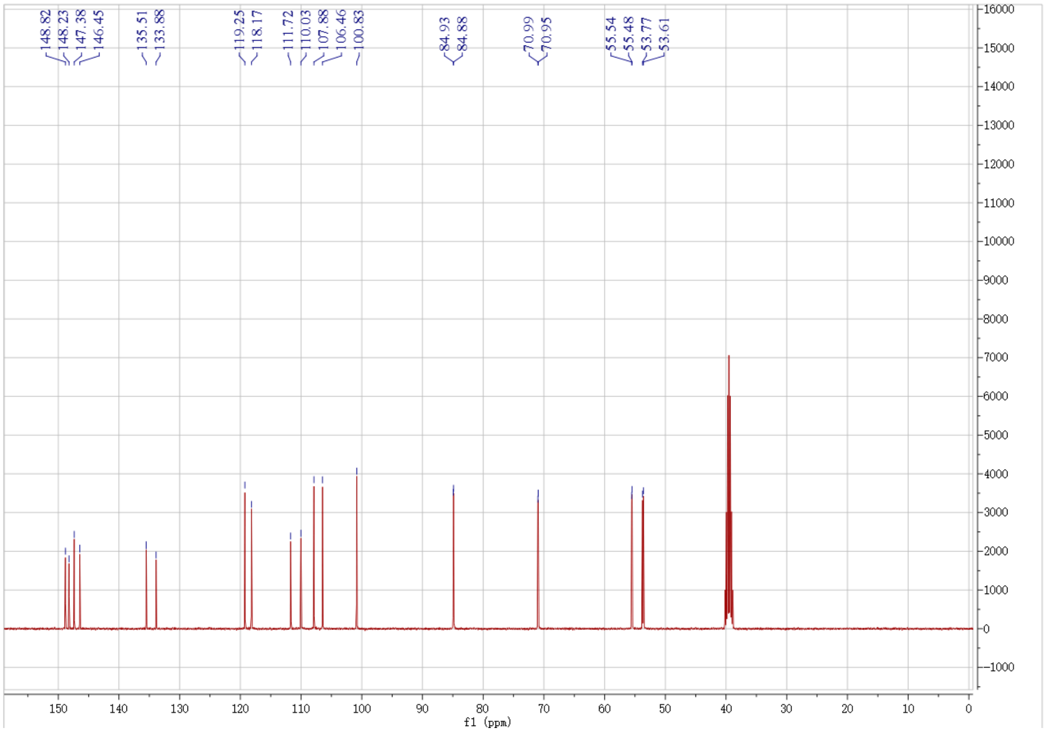

Figure S11.^{13}C NMR (100 MHz, DMSO-d_6) spectrum of **4**

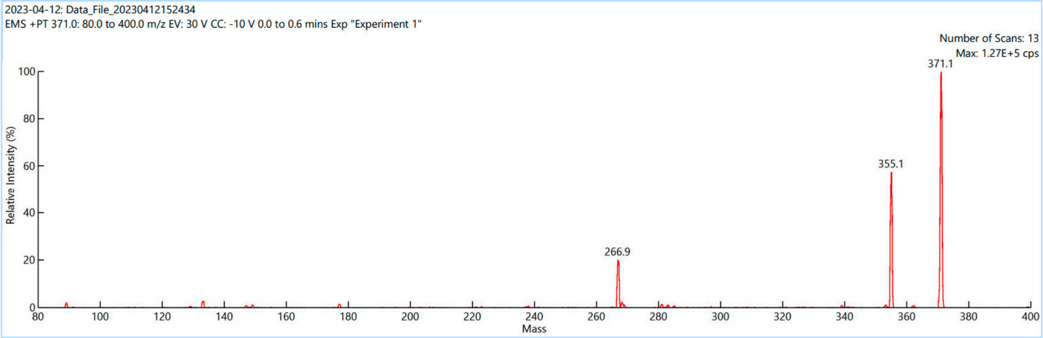

Figure S12. ESI-MS spectrum of **4**

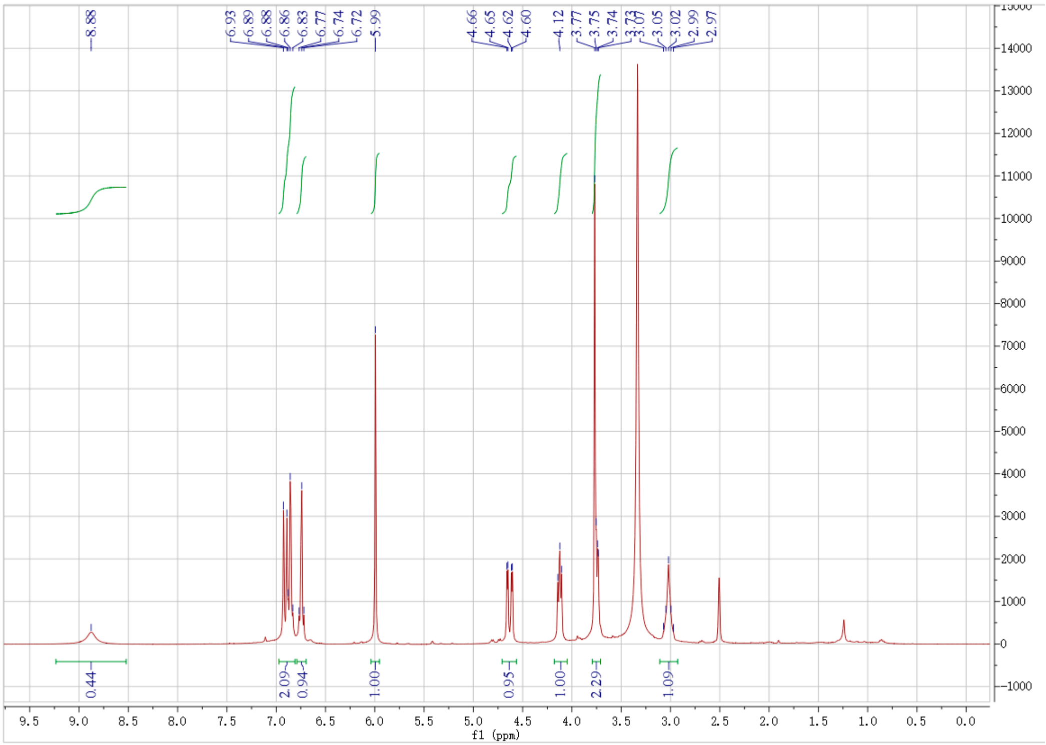

Figure S13. ^{1}H NMR (400 MHz, DMSO-d_6) spectrum of **5**

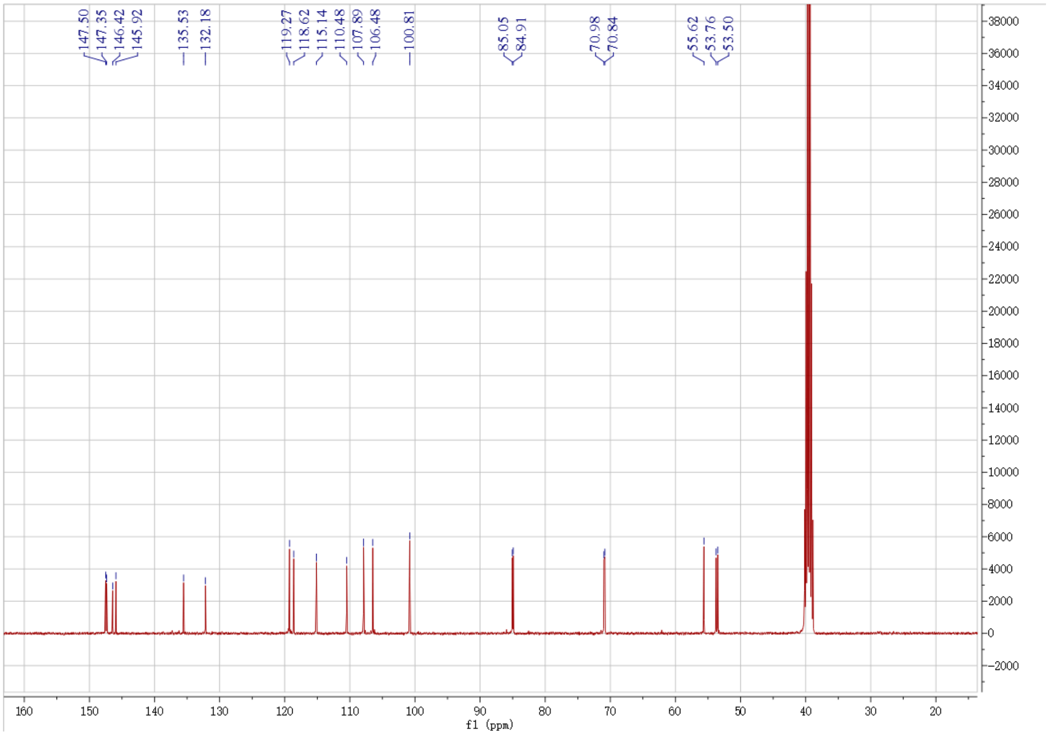

Figure S14.^{13}C NMR (100 MHz, DMSO-d_6) spectrum of **5**

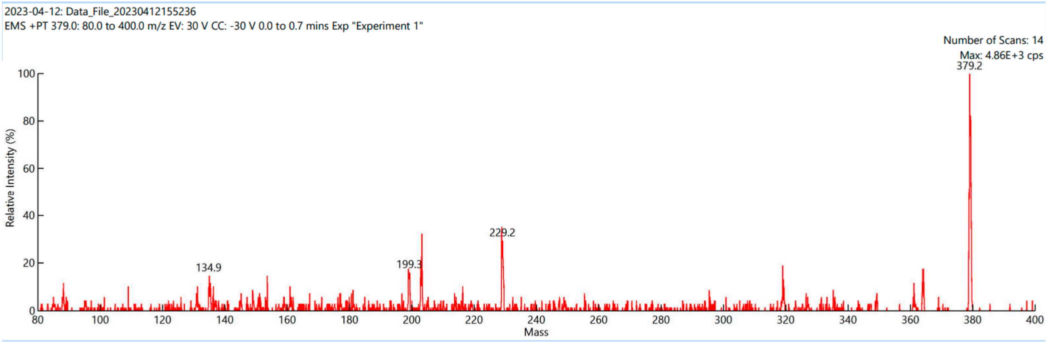

Figure S15. ESI-MS spectrum of **5**

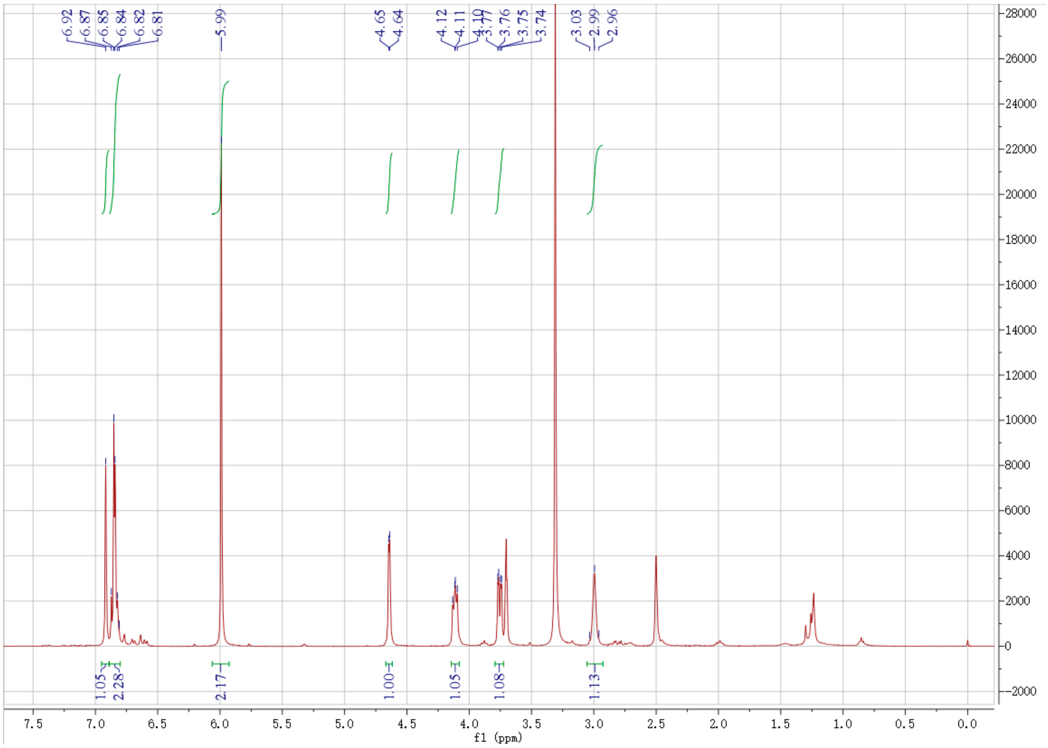

Figure S16. ^{1}H NMR (400 MHz, DMSO-d_6) spectrum of **6**

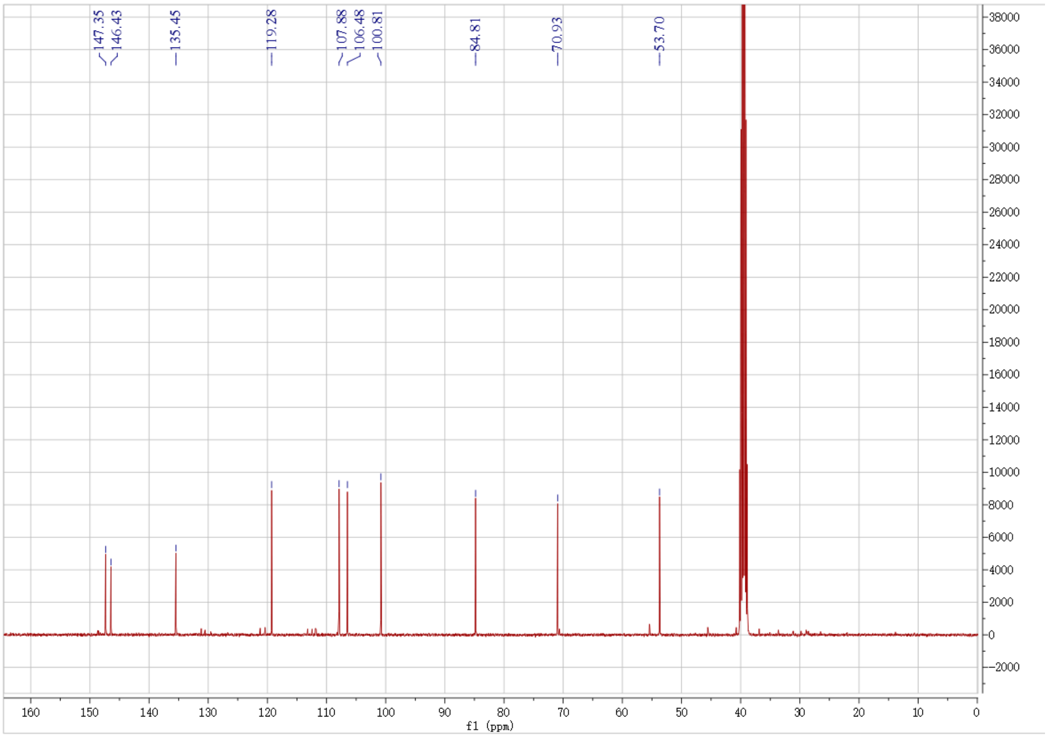

Figure S17.^{13}C NMR (100 MHz, DMSO-d$_6$) spectrum of **6**

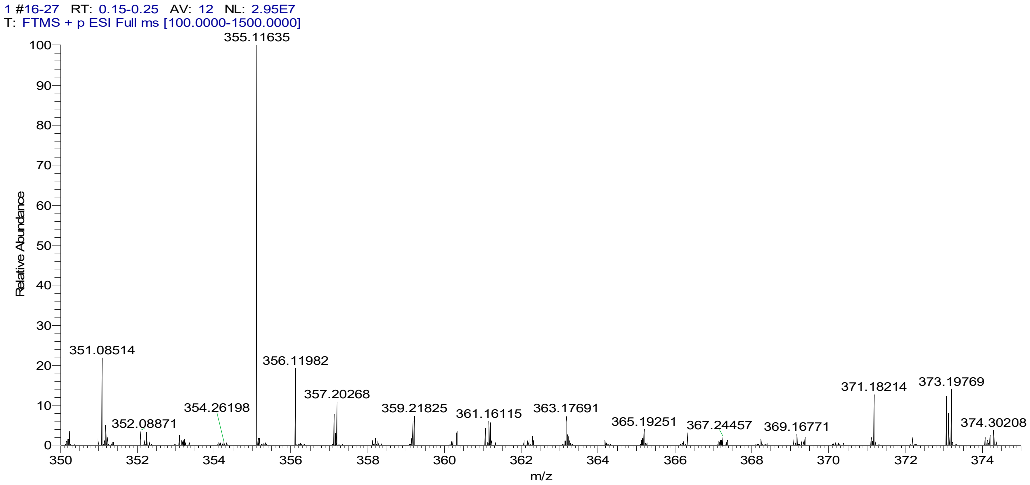

Figure S18. HRESI-MS spectrum of **6**

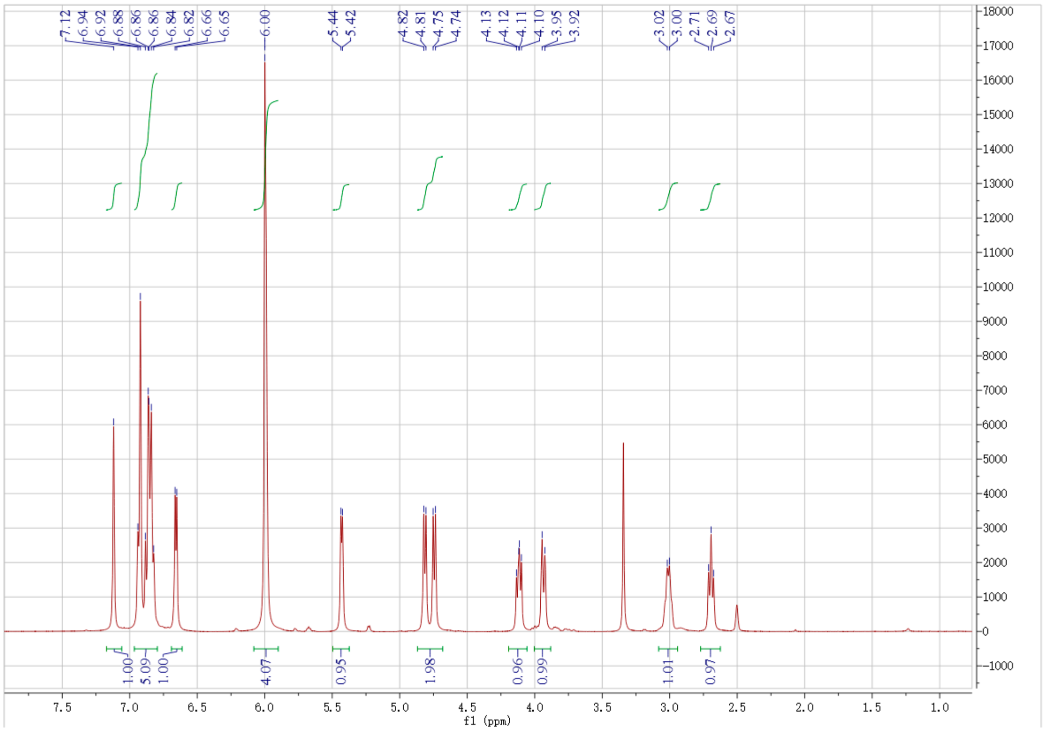

Figure S19. ^{1}H NMR (400 MHz, DMSO-d_6) spectrum of **7**

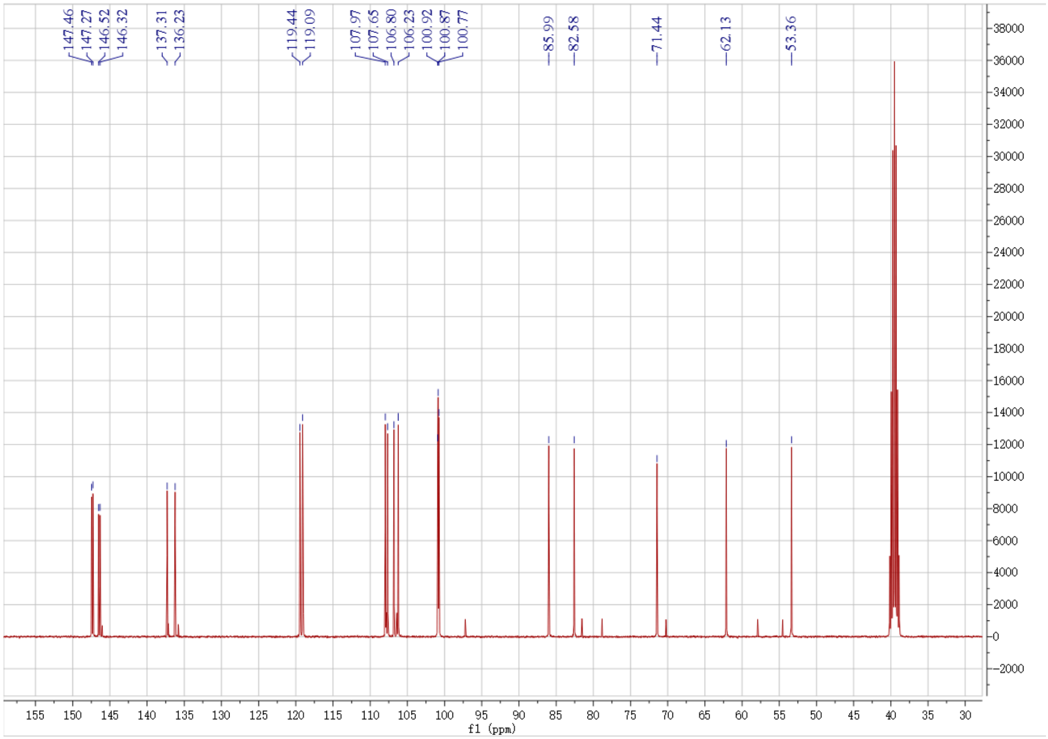

Figure S20.^{13}C NMR (100 MHz, DMSO-d_6) spectrum of **7**

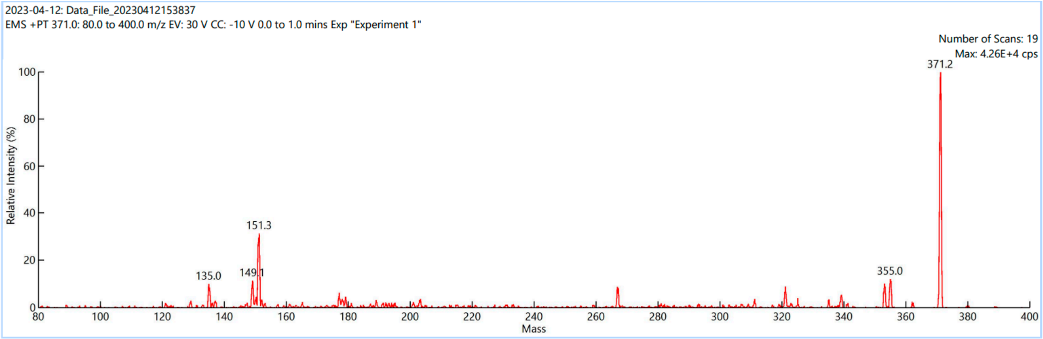

Figure S21. ESI-MS spectrum of **7**

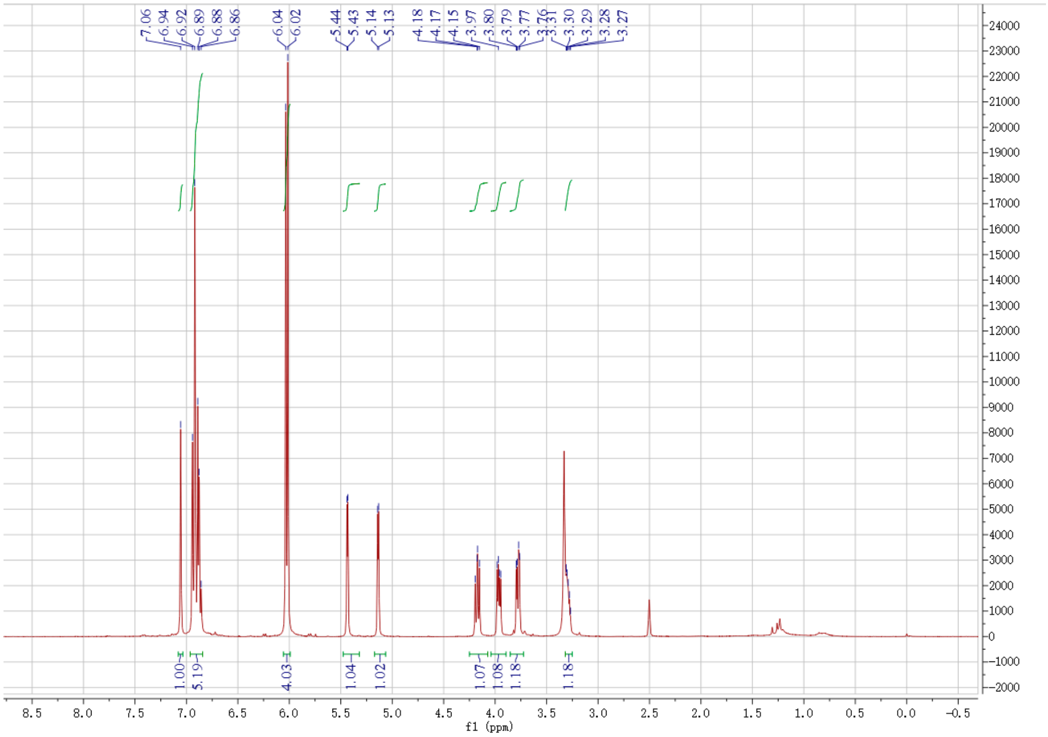

Figure S22. ^{1}H NMR (400 MHz, DMSO-d_6) spectrum of **8**

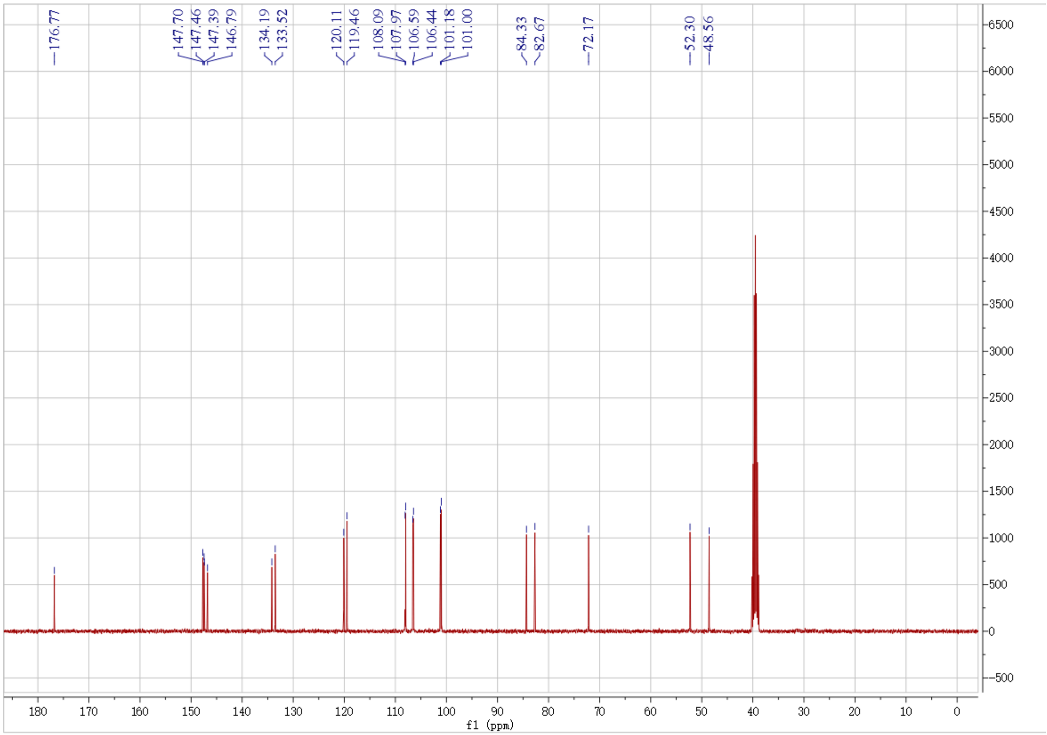

Figure S23.^{13}C NMR (100 MHz, DMSO-d_6) spectrum of **8**

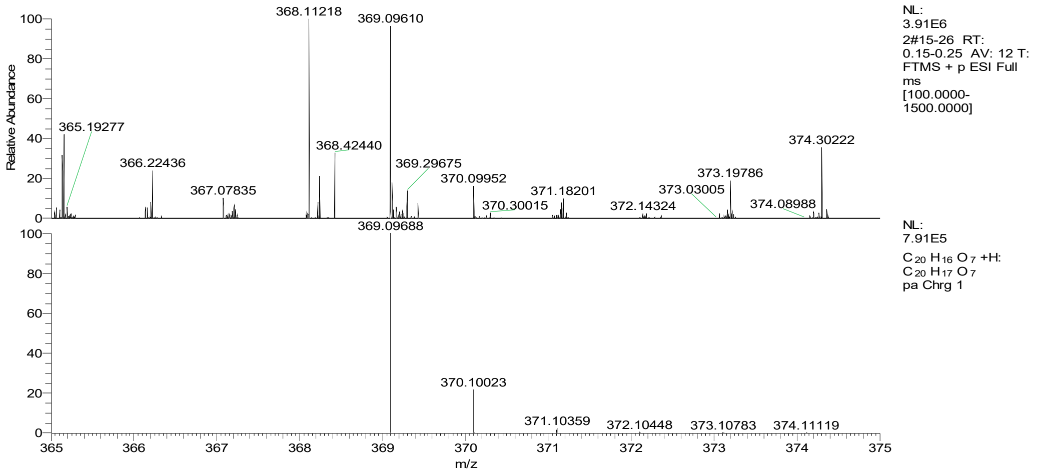

Figure S24. HRESI-MS spectrum of **8**

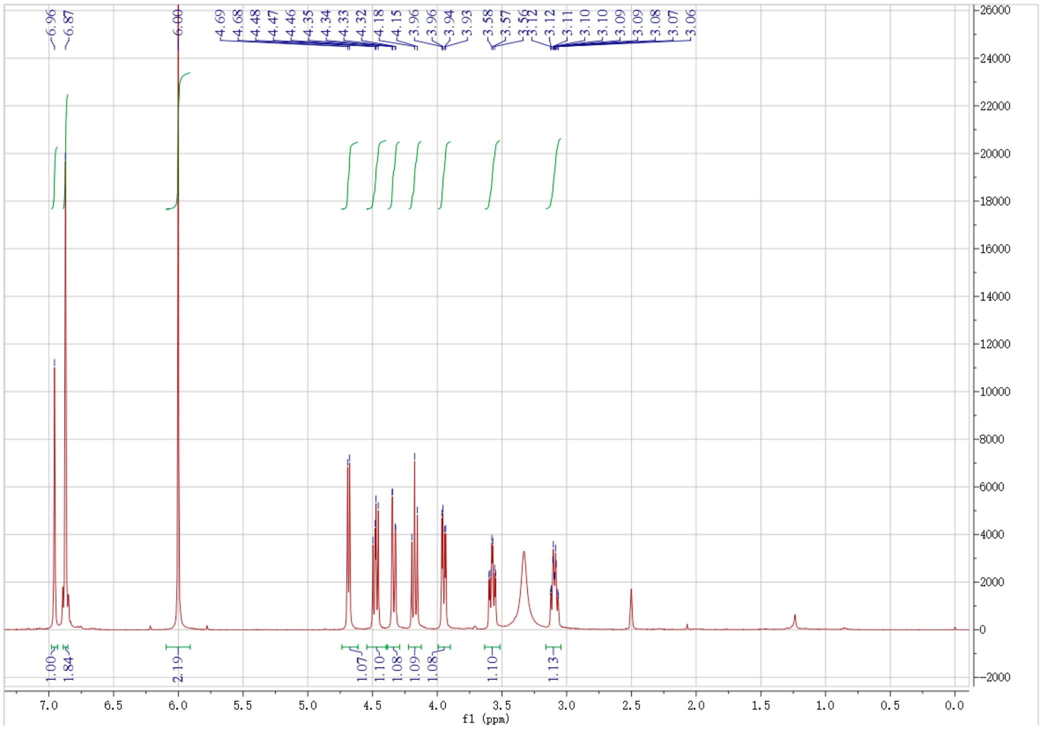

Figure S25. ^{1}H NMR (400 MHz, DMSO-d_6) spectrum of **9**

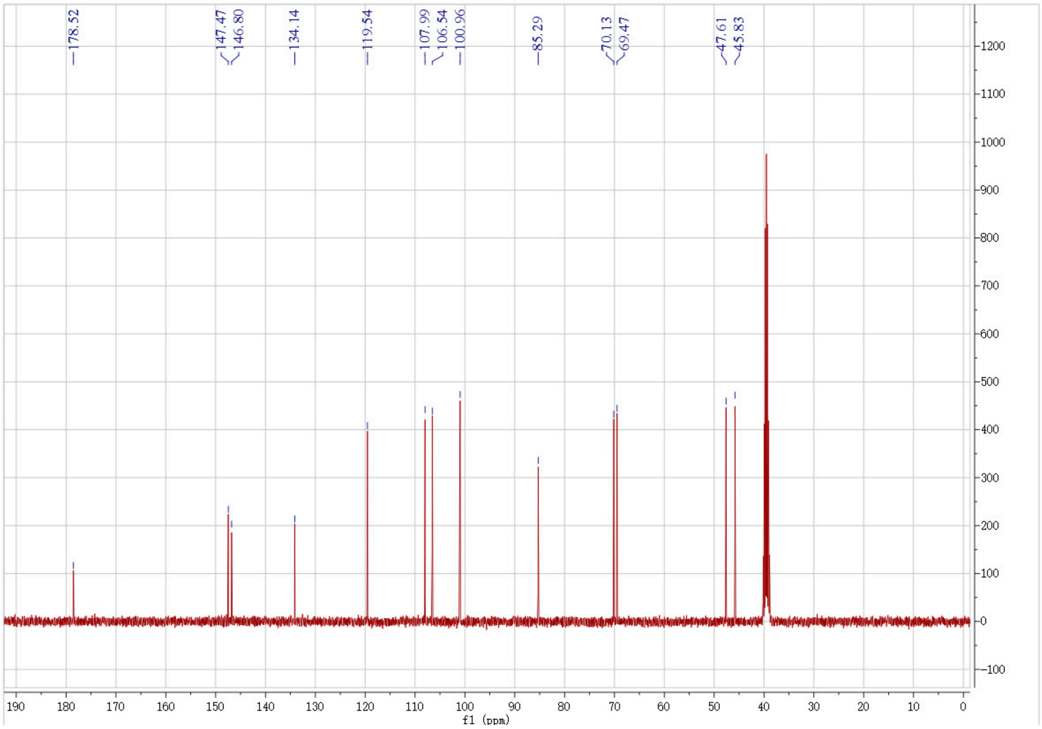

Figure S26.^{13}C NMR (100 MHz, DMSO-d$_6$) spectrum of **9**

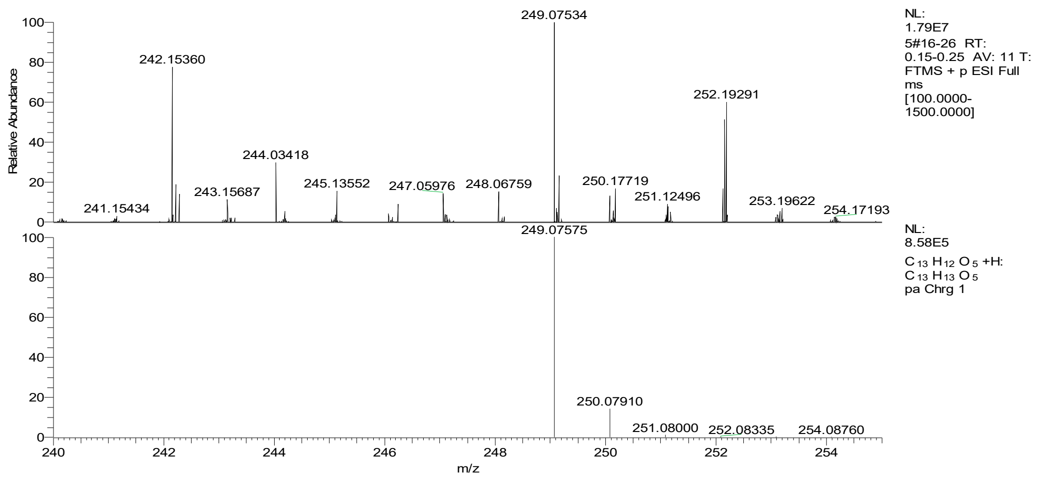

Figure S27. HRESI-MS spectrum of **9**

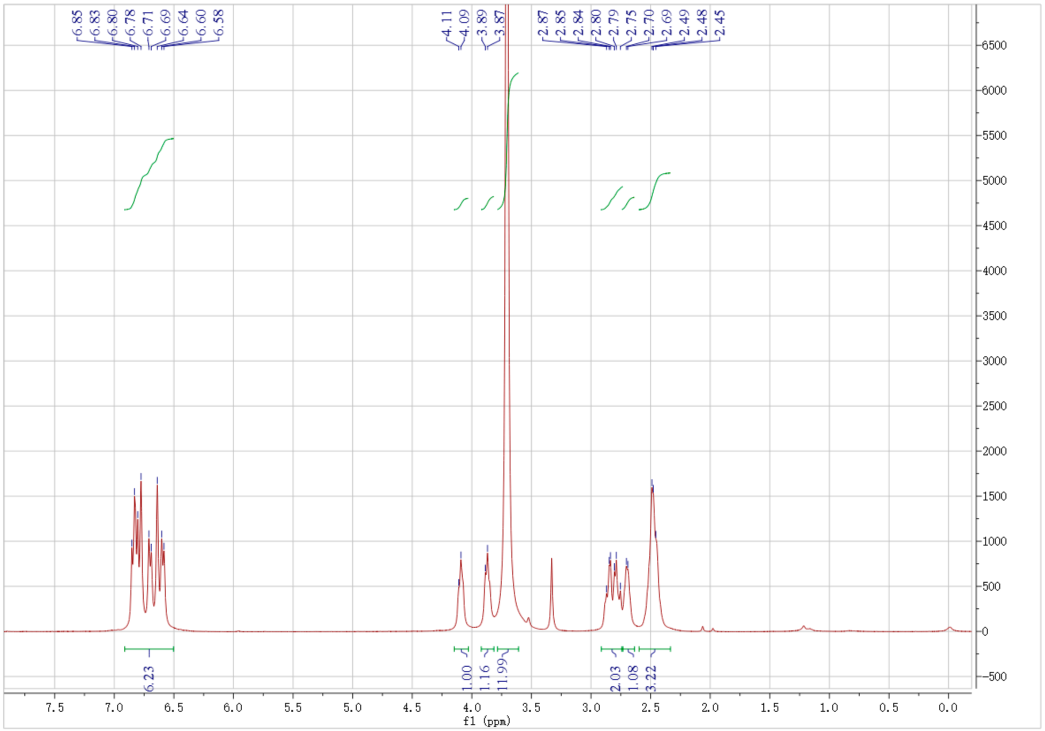

Figure S28. ^{1}H NMR (400 MHz, DMSO-d_6) spectrum of **10**

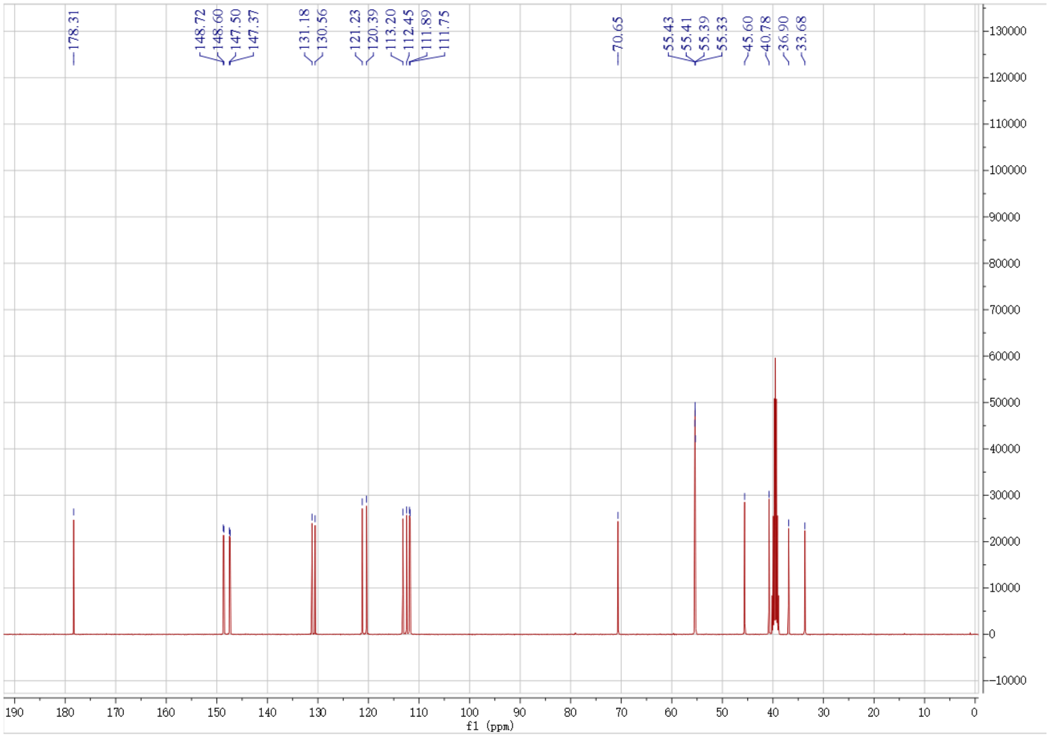

Figure S29.^{13}C NMR (100 MHz, DMSO-d_6) spectrum of **10**

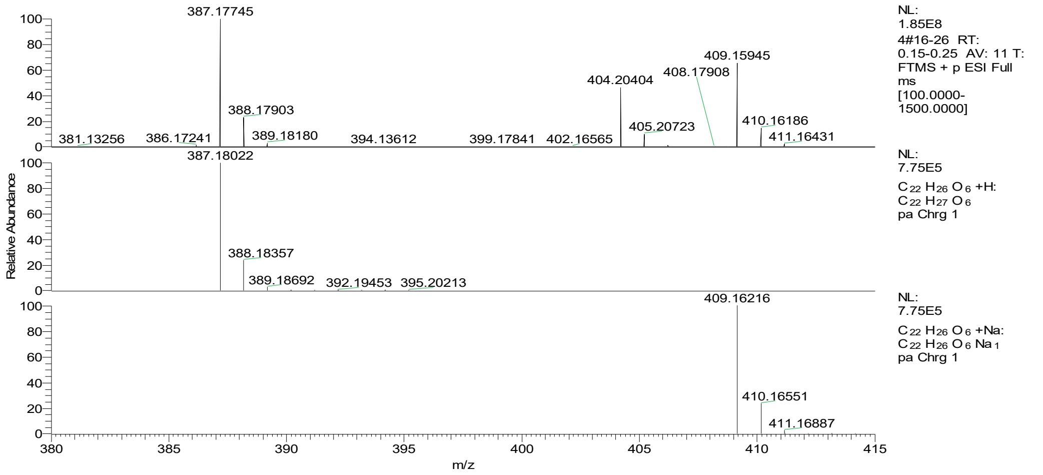

Figure S30. HRESI-MS spectrum of **10**

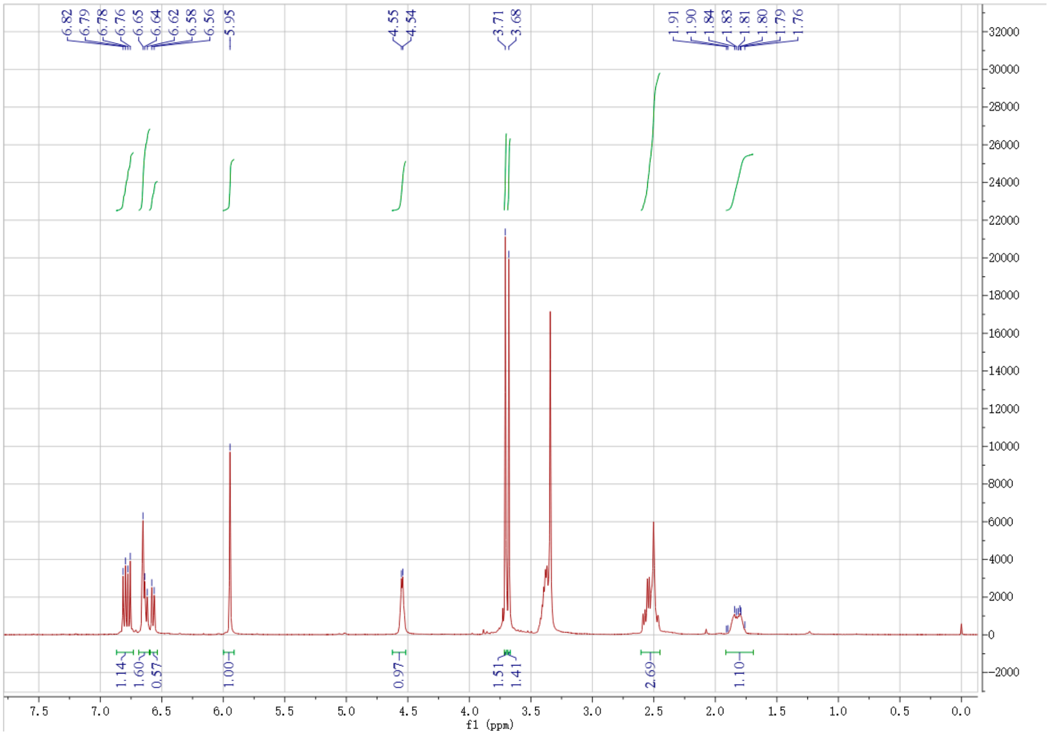

Figure S31. ^{1}H NMR (400 MHz, DMSO-d_6) spectrum of **11**

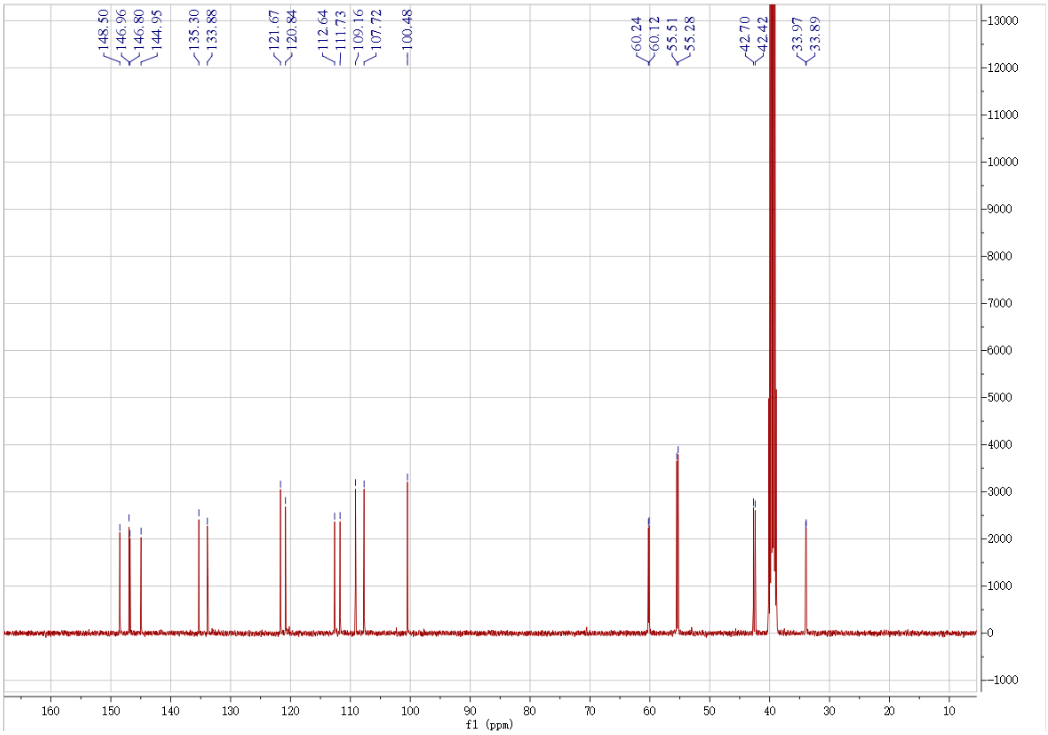

Figure S32.^{13}C NMR (100 MHz, DMSO-d_6) spectrum of **11**

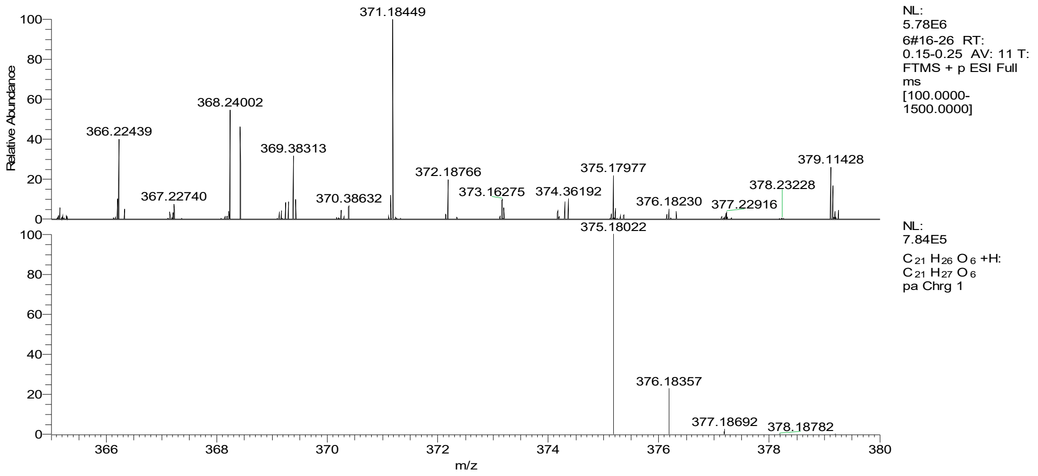

Figure S33. HRESI-MS spectrum of **11**

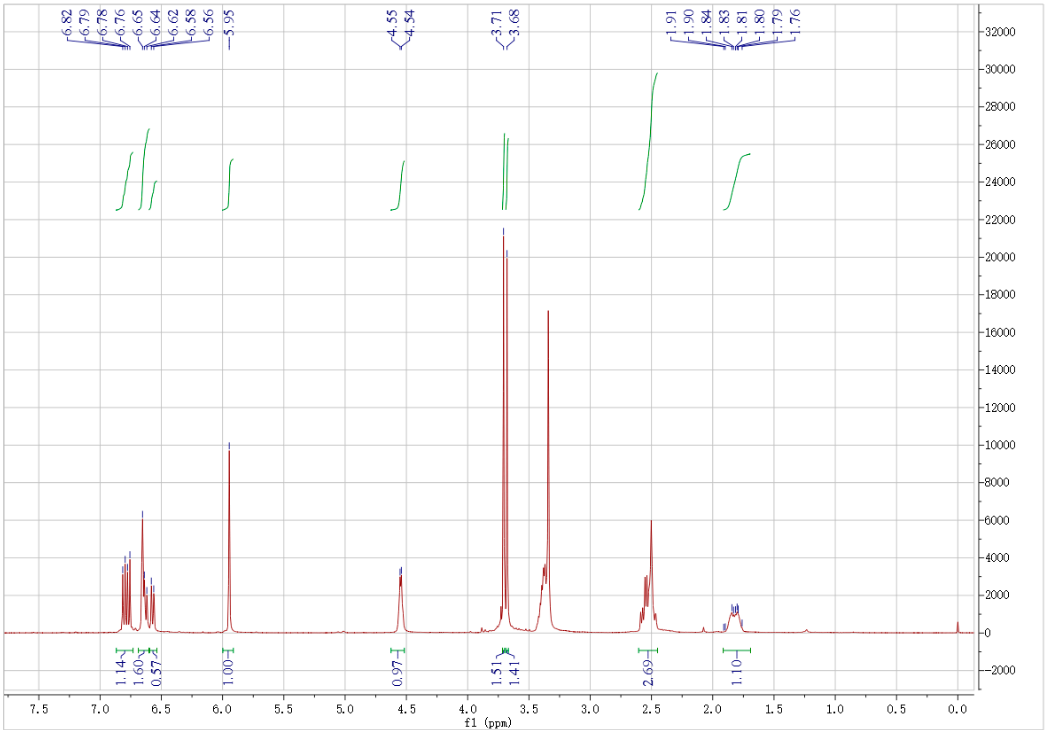

Figure S34. ^{1}H NMR (400 MHz, DMSO-d_6) spectrum of **12**

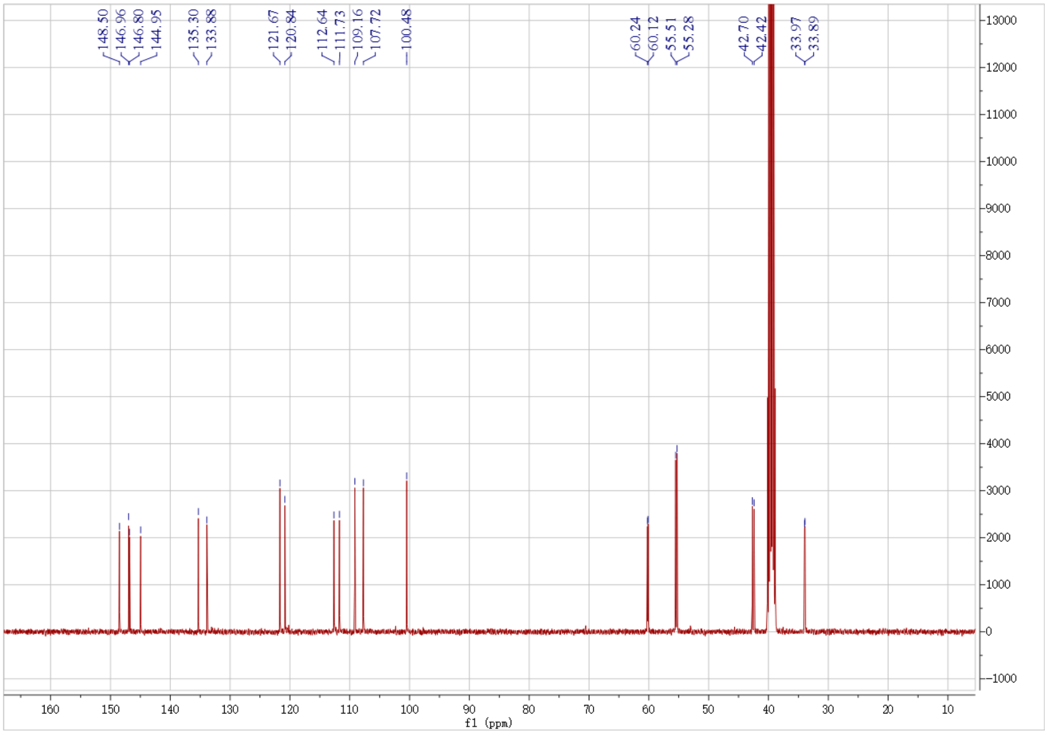

Figure S35.^{13}C NMR (100 MHz, DMSO-d_6) spectrum of **12**

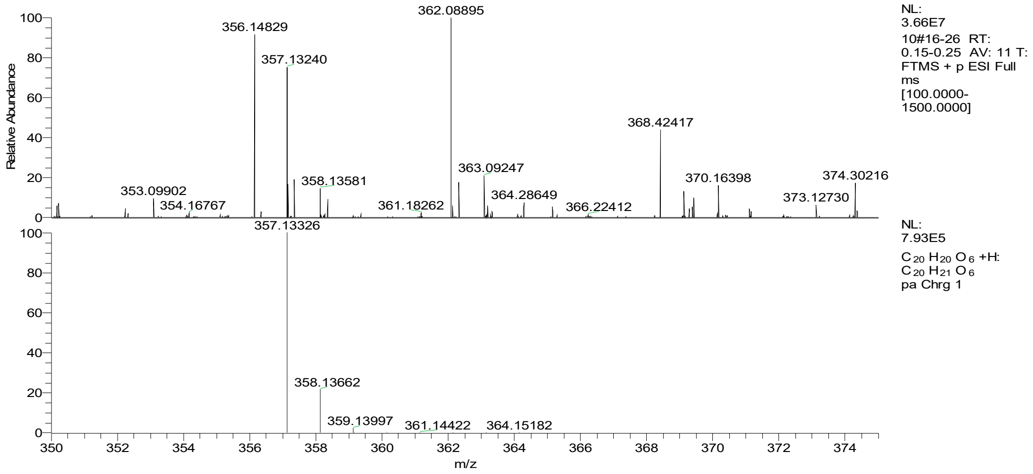

Figure S36. HRESI-MS spectrum of **12**

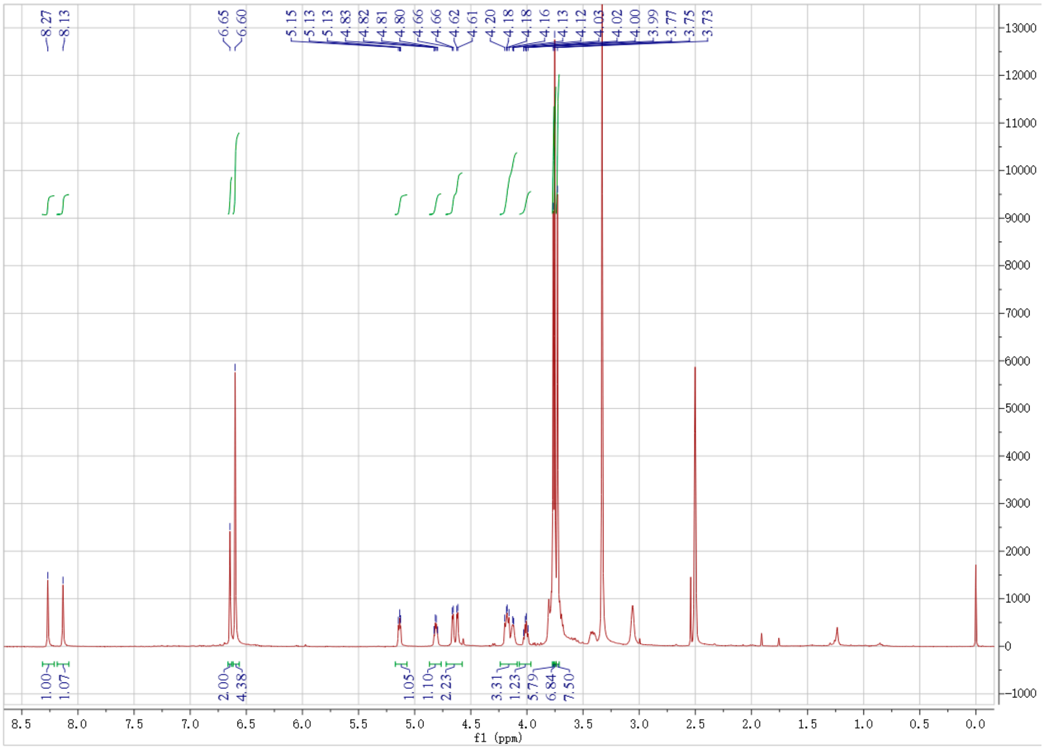

Figure S37. ^{1}H NMR (400 MHz, DMSO-d_6) spectrum of **13**

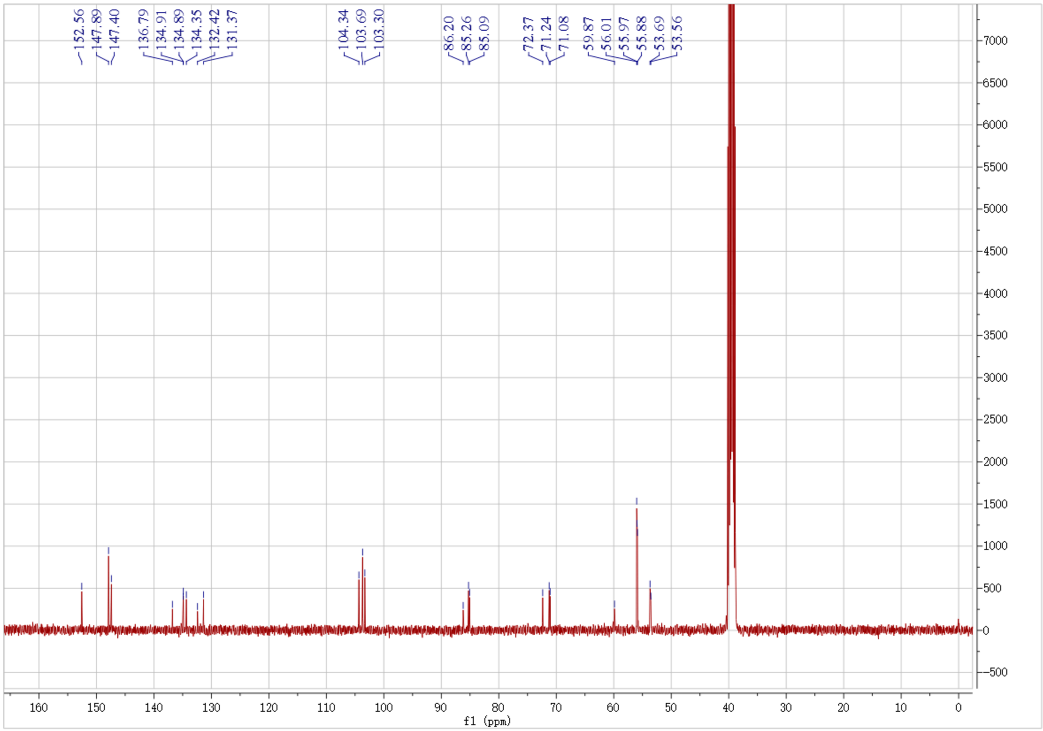

Figure S38.^{13}C NMR (100 MHz, DMSO-d_6) spectrum of **13**

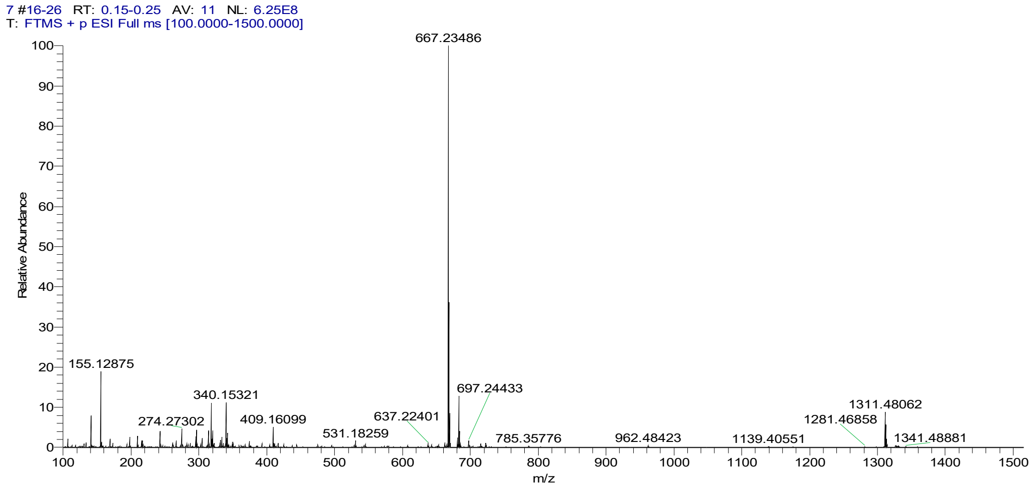

Figure S39. HRESI-MS spectrum of **13**

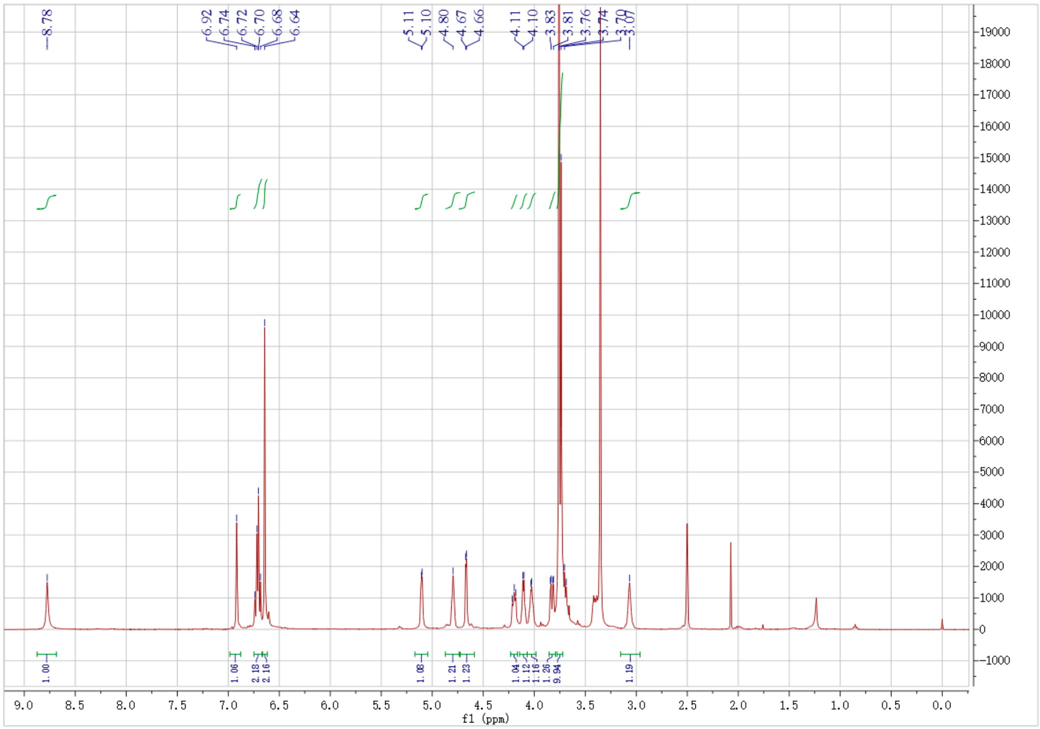

Figure S40. ^{1}H NMR (400 MHz, DMSO-d_6) spectrum of **14**

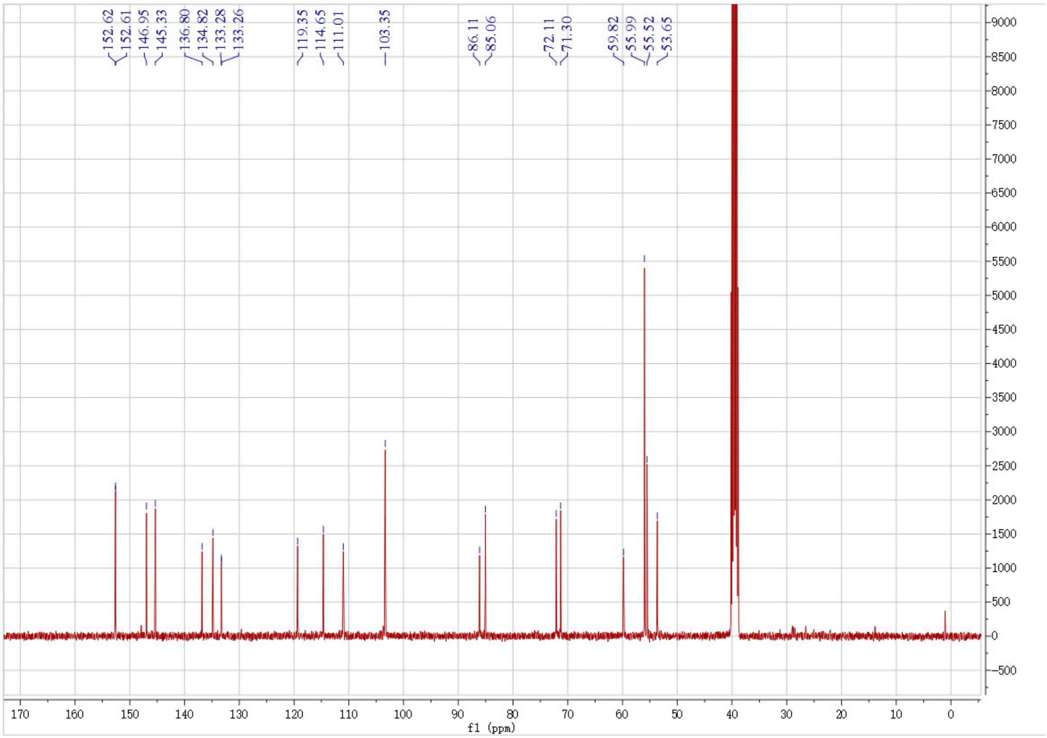

Figure S41.^{13}C NMR (100 MHz, DMSO-d_6) spectrum of **14**

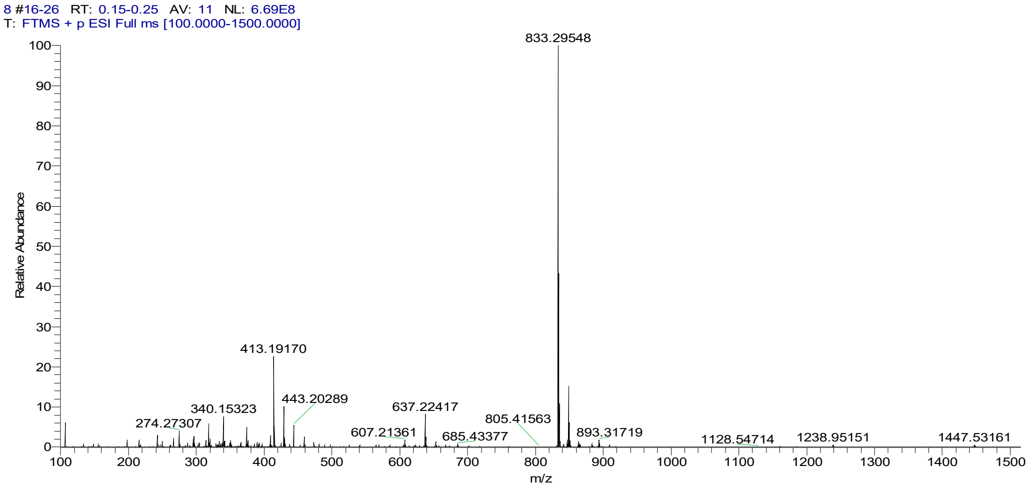

Figure S42. HRESI-MS spectrum of **14**

	Compound 1			
NO.	^{1}H NMR data(Exp)	^{1}HNMR data(Ref 5)	^{13}C NMR data(Exp)	^{13}C NMR data(Ref 5)
3,5	_	_	147.3	147.2
3'	_	_	146.9	146.7
4'	5.65 (1H, s, OH)	5.59 (1 H, s, OH)	145.4	145.3
4	5.54 (1H, OH)	5.48 (1 H, OH)	134.4	134.3
1'	_	_	133	132.9
1	_	_	132.3	132.1
6'	6.88 (1 H, d, J = 2.0 Hz)	6.89 (1 H, d, J = 2.1 Hz)	119.1	118.9
5'	6.82 (1H, dd, J = 8.1, 1.8 Hz)	6.82 (1 H, dd, J = 8.2 and 1.8 Hz)	114.4	114.3
2'	6.90 (1H, d, J = 2.0 Hz)	6.90 (1 H, d, J = 2. I Hz)	108.8	108.6
2,6	6.59 (2H, s)	6.59 (2H, s)	102.9	102.7
7	4.74 (2H, dd, J = 11.4, 4.4 Hz)	4.75 (2H, d, J=4.2 Hz)	86.3	86.1
9'			86	85.8
9	4.36–4.17 (2H, m); 3.84–3.77 (2H, m)	4.27 (2H, m); 3.88 (2H, m)	72	71.9
7'			71.8	71.6
3,5-OCH$_3$	3.90 (9H, s, C-3, 3', 5-OCH3)	3.90 (9H, s, C-3, 3', 5-OCH3)	56.5	56.4
3'-OCH$_3$			56.1	56
8	3.16–2.99 (2H, m)	3.10 (2H, m)	54.5	54.4
8'			54.2	54.1

	Compound 2			
NO.	^{1}H NMR data(Exp)	^{1}HNMR data(Ref 6)	^{13}C NMR data(Exp)	^{13}C NMR data(Ref 6)
3,5	_	_	147.1	147.2
3'	_	_	147.1	147.2
4'	_	_	134.3	134.4
4	_	_	134.3	134.4
1'	_	_	132	132.1
1	_	_	132	132.1
6'	6.57 (1H, s)	6.55 (1H, s)	102.7	102.8
5'	_	_	147.1	147.2
2'	6.57 (1H, s)	6.55 (1H, s)	102.7	102.8
2,6	6.57 (2H, s)	6.55 (2H, s)	102.7	102.8
7	4.72 (2H, d, J=4.2 Hz)	4.70 (2H, d, J=4.2 Hz)	86	86
9'			86	86
9	4.27 (2H, dd, J=9.0, 6.7 Hz); 3.87 (2H, dd, J=9.0, 3.4 Hz)	4.25 (2H, dd, J=9.0, 6.7 Hz); 3.88 (2H, dd, J=9.0, 3.4 Hz)	71.7	71.7
7'			71.7	71.7
3,5-OCH$_3$	3.84 (12H, s)	3.84 (12H, s)	56.3	56.3
3',5'-OCH$_3$			56.3	56.3
8	3.09 (2H, s)	3.08 (2H, s)	54.2	54.3
8'			54.2	54.3

	Compound 3			
NO.	^{1}H NMR data(Exp)	^{1}HNMR data(Ref 7)	^{13}C NMR data(Exp)	^{13}C NMR data(Ref 7)
3	_	_	146.9（C-3）	146.7（C-3）
5	6.81-6.90 (1H, m)	6.79-6.88 (1H, m)	114.4 (C-5)	114.2 (C-5)
3'	_	_	146.9	146.7
4'	5.66 (1H, s)	5.70 (1H, s)	145.4	145.2
4			145.4	145.2
1'	_	_	133.1	132.8
1	_	_	133.1	132.8
6'	6.81-6.90 (5H, m)	6.79-6.88 (5H, m)	119.1	118.9
5'			114.4	114.2
2'			108.8	108.6
2			108.8(C-2)	108.6(C-2)
6			119.1(C-6)	118.9(C-6)
7	4.74 (2H, d, J = 4.0 Hz)	4.71 (2H, d, J = 4.4 Hz)	86	85.8
9'			86	85.8
9	4.25 (2H, dd, J = 8.8, 4.4 Hz, H-7'a, 9a); 3.87 (2H, dd, J = 8.8, 4.4 Hz, H-7'b, 9b)	4.23 (2H, dd, J = 8.8, 4.4 Hz, H-7'a, 9a); 3.85 (2H, dd, J = 8.8, 4.4 Hz, H-7' b, 9b)	71.8	71.6
7'			71.8	71.6
3-OCH_3	3.90 (6H, s)	3.87 (6H, s)	56.1	55.9
3'-OCH_3			56.1	55.9
8	3.10 (2H, m, H-8, 8')	3.09 (2H, m, H-8, 8')	54.3	54.1
8'			54.3	54.1

	Compound 4			
NO.	^{1}H NMR data(Exp)	^{1}HNMR data(Ref 8)	^{13}C NMR data(Exp)	^{13}C NMR data(Ref 8)
3	_	_	147.4	148.1
5	7.06 – 6.76 (1H, m)	6.88-6.76 (1H, m)	111.7	111
3'	_	_	148.8	149.3
4'	5.66 (1H, s)	5.70 (1H, s)	148.2	148.7
4			146.4	147.2
1'	_	_	133.9	133.5
1	_	_	135.5	135.2
6'	7.06 – 6.76 (6H, m)	6.88-6.76 (5H, m)	118.2	118.4
5'			110	109.2
2'			106.5	106.7
2			107.9	108.3
6			119.3	119.5
7	4.66 (2H, d, J = 4.8 Hz)	4.71 (2H, t, J = 5.6 Hz)	84.9	85.9
9'			70.9	71.8
9	4.16-4.12 (2H, m, H-9a, 7'a); 3.78 (2H, d, J = 4.0 Hz, H-9b, 7'b)	4.22 (2H, m, H-9a, 7'a); 3.84 (2H, m, H-7'b, 9b)	71	71.9
7'			85	85.9
3'-OCH_3	3.74 (3H, s)	3.85 (3H, s)	55.5	56.1
4'-OCH_3	3.76 (3H, s)	3.87 (3H, s)	55.5	56
8	3.10 – 2.94 (2H, m, H-8, 8')	3.06 (2H, m, H-8, 8')	53.8	54.4
8'			53.6	54.3

	Compound 5			
NO.	1H NMR data(Exp)	1HNMR data(Ref 9)	13C NMR data(Exp)	13C NMR data(Ref 9)
3	_	_	147.5	147.5
5	6.95-6.80 (1H, m)	6.94-6.75 (1H, m)	115.1	115.1
3'	_	_	147.3	147.4
4'			146.4	146.4
4			145.9	145.9
1'	_	_	135.5	135.5
1	_	_	132.2	132.1
6'	6.95-6.80 (1H, m)	6.94-6.75 (5H, m)	119.3	119.3
5'	6.79-6.67 (1H, m)		107.9	107.9
2'	6.95-6.80 (2H, m)		106.5	106.5
2			110.5	110.4
6	6.79-6.67 (1H, m)		118.6	118.6
7	4.63 (2H, dd, J = 9.5, 4.4 Hz, H-7, 9’)	4.72 (2H, dd, J = 9.5, 4.4 Hz, H-7, 9’)	85.1	85
9'			84.9	84.9
9	4.19-4.04 (2H, m, H-9a, 7’a); 3.76-3.71 (2H, m, H-9b, 7’b)	4.22 (2H, m, H-9a, 7’a); 3.84 (2H, m, H-7’b, 9b)	71	71
7'			70.8	70.8
3-OCH_3	3.77 (3H, s)	3.89 (3H, s)	55.6	55.6
8	3.09-2.93 (2H, m)	3.25-2.90 (2H, m)	53.5	53.5
8'			53.8	53.8

	Compound 6			
NO.	1H NMR data(Exp)	1HNMR data(Ref 10)	13C NMR data(Exp)	13C NMR data(Ref 10)
3	_	_	146.4	146.7
5	6.89-6.79 (1H, m)	6.8 (1H, m)	106.5	106.4
3'	_	_	146.4	146.7
4'			147.4	147.9
4			147.4	147.9
1'	_	_	135.4	134.9
1	_	_	135.4	134.9
6'	6.89-6.79 (1H, m)	6.8 (5H, m)	119.3	119.2
5'	6.89-6.79 (1H, m)		106.5	106.4
2'	6.90 (2H, s)		107.9	108.1
2			107.9	108.1
6	6.89-6.79 (1H, m)		119.3	119.2
7	4.64 (2H, d, J = 3.6 Hz, H-7, 9’)	4.7 (2H, d, J = 4.1 Hz, H-7, 9’)	84.8	85.7
9'			84.8	85.7
9	4.11 (2H, dd, J = 6.7, 8.6 Hz, H-9a, 7’a); 3.76 (2H, dd, J = 2.9, 9.0 Hz, H-9b, 7’b)	4.2 (2H, dd, J = 7.0, 9.3 Hz, H-9a, 7’a); 3.8 (2H, dd, J = 3.5, 9.3 Hz, H-9b, 7’b)	70.9	71.6
7'			70.9	71.6
8	3.06-2.91(2H, m)	3.1(2H, m)	53.7	54.2
8'			53.7	54.2
10	5.99 (4H, s)	5.9 (4H, s)	100.8	101.1
10'			100.8	101.1

	Compound7			
NO.	^{1}H NMR data(Exp)	^{1}HNMR data(Ref 11)	^{13}C NMR data(Exp)	^{13}C NMR data(Ref 11)
3	_	_	147.5	147.5
5	6.94-6.82 (1H, m)	6.8-7.1 (1H, m)	106.8	106.8
3'	_	_	147.3	147.3
4'			146.3	146.3
4			146.5	146.5
1'	_	_	136.2	136.3
1	_	_	137.3	137.3
6'	6.94-6.82 (2H, m)	6.8-7.1 (5H, m)	119.1	119.1
5'			106.2	106.2
2'	6.94-6.82 (1H, m)		107.7	107.7
2	7.12 (1H, s)		108	108
6	6.94-6.82 (1H, m))		119.4	119.5
7	4.78 (2H, dd, J = 27.9, 6.6 Hz)	4.79 (1H, d, J = 6.6 Hz, H-7); 4.71 (1H, d, J = 6.6 Hz, H-9')	82.6	82.5
9'			71.4	71.4
9	5.43 (1H, d, J = 4.6 Hz)	5.40 (1H, s)	100.9	100.9
7'	4.12 (1H, dd, J = 8.2, 6.4 Hz, H-7'a), 3.94 (1H, d, J = 8.3 Hz, H-7'b)	4.09 (1H, dd, J = 9, 6 Hz, H-7'a), 3.91 (1H, d, J = 6.6 Hz, H-7'b)	86	86
8	2.69 (1H, m, H-8)	2.66 (1H, m, H-8)	62.1	62.2
8'	3.30-2.97(1H, m, H-8')	2.98 (1H, m, H-8')	53.4	53.4

	Compound8			
NO.	^{1}H NMR data(Exp)	^{1}HNMR data(Ref 12)	^{13}C NMR data(Exp)	^{13}C NMR data(Ref 12)
3	_	_	147.7	148.4
5	6.96-6.82 (1H, m)	6.76-6.87 (1H, m)	106.6	106
3'	_	_	147.5	147.3
4'	_	_	146.8	146.8
4	_	_	147.4	147.4
1'	_	_	133.5	133.1
1	_	_	134.2	134.4
6'	6.96-6.82 (3H, m)	6.76-6.87 (5H, m)	119.5	118.8
5'			106.4	105.7
2'			108	108.4
2	7.06 (1H, s)		108.1	108.6
6	6.96-6.82 (1H, m)		120.1	119
7	5.44 (1H, d, J = 3.6 Hz)	5.32 (1H, d, J = 3.6 Hz)	84.3	84.4
9'	5.14 (1H, d, J = 3.8 Hz)	5.30 (1H, d, J = 3.6 Hz)	82.7	83.4
9			176.8	176.6
7'	4.17 (1H, dd, J = 9.2, 7.4 Hz, H-7'a), 3.96 (1H, d, J = 9.4, 4.5 Hz, H-7'b)	4.32 (1H, dd, J = 9.2, 6.8 Hz, H-7'a), 4.02 (1H, d, J = 9.4, 4.8 Hz, H-7'b)	72.2	72.7
8	3.78 (1H, dd, J = 9.3, 3.8 Hz)	3.42 (1H, dd, J = 9.2, 3.6 Hz,)	52.3	53.3
8'	3.31-3.24(1H, m)	3.2(1H, m)	48.6	50
10	6.03 (4H, d, J = 8.0 Hz, H-10, 10')	5.98 (1H, s); 5.96(1H, s)	101.2	101.5
10'			101	101.2

	Compound 9			
NO.	^{1}H NMR data(Exp)	^{1}HNMR data(Ref 13)	^{13}C NMR data(Exp)	^{13}C NMR data(Ref 13)
3	_	_	147.5	148.3
5	6.87 (1H, s)	6.82 (1H, s)	108	108.4
4	_	_	146.8	147.8
1	_	_	134.1	132.8
2	6.96 (1H, s)	6.86 (1H, s)	106.5	106.4
6	6.87 (1H, s)	6.81 (1H, s)	119.5	119.6
7	4.69 (1H, d, J = 6.3 Hz)	4.62 (1H, d, J = 7.0 Hz)	85.3	86.1
9'	4.48 (1H, dd, J = 9.4, 6.9 Hz, H-9'a); 4.18 (1H, t, J = 8.6 Hz, H-9'b)	4.51 (1H, m, H-9'a); 4.21 (1H, dd, J = 3.5 Hz, J = 9.5 Hz, H-9'b)	70.1	70.1
9	_	_	178.5	178.1
7'	4.34 (1H, dd, J = 9.5, 1.8 Hz, H-7'a); 3.95 (1H, dd, J = 9.0, 3.2 Hz, H-7'b)	4.38 (1H, d, J = 7.0 Hz, H-7'a), 4.35 (1H, m, H-7'b)	69.5	69.8
8	3.09 (1H, dtd, J = 8.6, 6.8, 1.8 Hz, H-8)	3.10 (1H, m)	45.8	46
8'	3.57 (1H, td, J = 8.7, 3.2 Hz)	3.45 (1H, td, J = 9.5, 3.5Hz)	47.6	48.4
10	6.00 (2H, s)	5.99 (2H, s)	101	101.3

	Compound10			
NO.	^{1}H NMR data(Exp)	^{1}HNMR data(Ref 14)	^{13}C NMR data(Exp)	^{13}C NMR data(Ref 14)
1	—	—	178.3	178
2	2.70 (1H, d, J = 5.0 Hz)	2.70 (1H, d, J = 5.0 Hz)	45.6	46.1
3	2.57 –2.36 (1H, m)	2.57 –2.36 (1H, m)	40.8	40.7
4	4.10 (1H, d, J = 7.2 Hz, H-4a), 3.88 (1H, d, J = 7.2 Hz, H-4b)	4.10 (1H, d, J = 7.2 Hz, H-4a), 3.88 (1H, d, J = 7.2 Hz, H-4b)	70.7	70.7
5	2.57 –2.36 (2H, m)	2.57 –2.36 (2H, m)	33.7	34.1
1'	—	—	131.2	130.3
2'	6.95 – 6.39 (1H, m)	6.95 – 6.39 (1H, m)	113.2	112.6
3'	—	—	148.7	148.9
4'			147.5	147.7
5'	6.95 – 6.39 (1H, m)	6.95 – 6.39 (1H, m)	111.9	112
6'	6.95 – 6.39 (1H, m)	6.95 – 6.39 (1H, m)	121.2	121.1
1''	—	—	130.6	130
2''	6.95 – 6.39 (1H, m)	6.95 – 6.39 (1H, m)	112.5	112.6
3''	—	—	148.6	148.9
4''			147.4	147.7
5''	6.95 – 6.39 (1H, m)	6.95 – 6.39 (1H, m)	111.8	111.4
6''	6.95 – 6.39 (1H, m)	6.95 – 6.39 (1H, m)	120.4	120.3
7	2.82 (2H, dt, J = 20.0, 11.3 Hz)	2.82 (2H, dt, J = 20.0, 11.3 Hz)	36.9	37.97
(3', 4', 3'', 4''-OCH3)	3.71 (12H, s)	3.71 (12H, s)	55.4	55.5

	Compound11			
NO.	^{1}H NMR data(Exp)	^{1}HNMR data(Ref 15)	^{13}C NMR data(Exp)	^{13}C NMR data(Ref 15)
1	—	—	135.3	134.3
2	6.84 – 6.53 (1H, m)	6.78 – 6.60 (1H, m)	107.7	108.1
3	—	—	147	147.6
4	—	—	145	145.8
5	6.84 – 6.53 (1H, m)	6.78 – 6.60 (1H, m)	109.2	109.3
6	6.84 – 6.53 (1H, m)	6.78 – 6.60 (1H, m)	121.7	121.8
7	2.60–2.42 (1H, m)	2.65 (1H, m)	34	35.9
8	1.92 – 1.72 (1H, m)	1.81 (1H, m)	55.5	55.9
9	3.44 –3.36 (2H, m)	3.51 (2H, m)	60.2	60.6
1'	—	—	133.9	133
2'	6.84 – 6.53 (1H, m)	6.78 – 6.60 (1H, m)	111.7	111.1
3'	—	—	148.5	148.9
4'			146.8	147.4
5'	6.84 – 6.53 (1H, m)	6.78 – 6.60 (1H, m)	112.6	112
6'	6.84 – 6.53 (1H, m)	6.78 – 6.60 (1H, m)	120.8	121
7'	2.60–2.42 (1H, m)	2.65 (1H, m)	33.9	35.8
8'	1.92 – 1.72 (1H, m)	1.81 (1H, m)	42.4	44.1
9'	3.44 –3.36 (2H, m)	3.51 (2H, m)	60.1	60.5
(3', 4'-OCH3)	3.71 (3H, s), 3.68 (3H, s)	3.84 (3H, s), 3.81 (3H, s)	55.5	55.8

	Compound12			
NO.	^{1}H NMR data(Exp)	^{1}HNMR data(Ref 16)	^{13}C NMR data(Exp)	^{13}C NMR data(Ref 16)
1	—	—	131.7	131.8
2	6.92 (1H, s)	6.93 (1H, s)	110.5	110.5
3	—	—	147.6	147.7
4	—	—	146.6	146.7
5	6.77 (1H, s)	6.76 (1H, s)	115.4	115.5
6	6.75 (1H, s)	6.76 (1H, s)	118.7	118.8
7	5.56 (1H, d, J = 6.7 Hz)	5.56 (1H, d, J = 6.6 Hz)	88.1	88.3
8	3.53 (1H, dd, J = 12.1, 6.0 Hz)	3.54 (1H, m)	52.4	52.5
9	3.73 – 3.62 (2H, m)	3.67 (2H, m)	62.7	62.8
1'	—	—	127.7	127.8
2'	7.32 (1H, s)	7.32 (1H, s)	112.6	112.6
3'	—	—	144.1	144.2
4'			150.7	150.8
5'	—	—	130.1	130.3
6'	7.32 (1H, s)	7.32 (1H, s)	118.9	118.8
7’	7.65 (1H, d, J = 15.7 Hz)	7.65 (1H, d, J = 16.0 Hz)	153.9	154.2
8’	6.80 (1H, d, J = 7.8 Hz)	6.80 (1H, d, J = 7.8 Hz)	126.1	126.2
9’	9.60 (1H, d, J = 7.8 Hz)	9.60 (1H, d, J = 7.8 Hz)	194	194.2
(3-OCH3)	3.75 (3H, s)	3.75 (3H, s)	55.7	55.7
(3'-OCH3)	3.84 (3H, s)	3.84 (3H, s)	55.8	55.9

	Compound13			
NO.	^{1}H NMR data(Exp)	^{1}HNMR data(Ref 17)	^{13}C NMR data(Exp)	^{13}C NMR data(Ref 17)
1	—	—	132.4	132.1
2	6.60 (1H, s)	6.59 (1H, s)	103.7	103
3	—	—	147.9	147.4
4	—	—	134.9	134.2
5	—	—	147.9	147.4
6	6.60 (1H, s)	6.59 (1H, s)	103.7	103
7	4.64 (1H, d, J = 3.7 Hz)	4.76 (1H, d, J = 3.4 Hz)	85.1	86
8	3.11 (1H, m)	3.11 (1H, m)	53.6	54.5
9	4.21 – 4.09 (2H, m)	4.12 (2H, m)	71.1	71.8
1'	—	—	136.8	137.9
2'	6.65 (1H, s)	6.64 (1H, s)	103.7	103
3'	—	—	152.6	153.6
4'	—	—	134.9	134.6
5'	—	—	152.6	153.6
6'	6.65 (1H, s)	6.64 (1H, s)	103.7	103
7’	4.21 – 4.09 (1H, m, H-7'a); 4.01 (1H, dd, J = 10.0, 5.8 Hz, H-7’b)	4.12 (2H, m)	71.2	71.8
8’	3.11 (1H, m)	3.11 (1H, m)	53.7	54.5
9’	4.64 (1H, d, J = 3.7 Hz)	4.76 (1H, d, J = 3.4 Hz)	85.3	86
1''	—	—	131.4	130.6
2''	6.60 (1H, s)	6.59 (1H, s)	104.3	103
3''	—	—	147.4	147.2
4''	—	—	134.3	134.5
5''	—	—	147.4	147.2
6''	6.60 (1H, s)	6.59 (1H, s)	103.3	102.8
7’'	5.17 – 5.09 (1H, m)	4.98 (1H, m)	72.4	72.2
8’'	4.81 (1H, dd, J = 7.8, 4.6 Hz)	4.81 (1H, dd, J = 7.8, 4.6 Hz)	86.2	86.1
9’'	3.93 (1H, m)	3.93 (1H, m)	59.9	60.6
(3, 5-OCH3)	3.77 (6H, s)	3.87 (6H, s)	56	56.5
(3', 5'-OCH3)	3.75 (6H, s)	3.89 (6H, s)	56	56.5
(3'', 5''-OCH3)	3.73 (6H, s)	3.90 (6H, s)	55.9	56.4

	Compound14			
NO.	^{1}H NMR data(Exp)	^{1}HNMR data(Ref 18)	^{13}C NMR data(Exp)	^{13}C NMR data(Ref 18)
1	—	—	134.8	134.8
2	6.64 (1H, s)	6.69 (1H, s)	103.3	103.3
3	—	—	152.6	152.6
4	—	—	136.8	136.8
5	—	—	152.6	152.6
6	6.64 (1H, s)	6.69 (1H, s)	103.3	103.3
7	4.67 (1H, d, J = 3.4 Hz)	4.67 (1H, d, J = 3.7 Hz)	85.1	85.1
8	3.12 – 2.99 (1H, m)	3.06 (1H, m)	53.6	53.7
9	4.03 (2H, d, J = 3.0 Hz, H-9a, 7’a), 3.85 – 3.79 (2H, m, H-9b, 7’b)	3.70 (2H, m)	71.3	71.3
1'	—	—	134.8	134.8
2'	6.64 (1H, s)	6.69 (1H, s)	103.3	103.3
3'	—	—	152.6	152.6
4'	—	—	136.8	136.8
5'	—	—	152.6	152.6
6'	6.64 (1H, s)	6.69 (1H, s)	103.3	103.3
7’	4.03 (2H, d, J = 3.0 Hz, H-9a, 7’a), 3.85 – 3.79 (2H, m, H-9b, 7’b),	3.70 (2H, m)	71.3	71.3
8’	3.12 – 2.99 (1H, m)	3.06 (1H, m)	53.6	53.7
9’	4.67 (1H, d, J = 3.4 Hz)	4.67 (1H, d, J = 3.7 Hz)	85.1	85.1
1''	—	—	133.3	133.3
2''	6.76 – 6.67 (1H, m)	6.72 (1H, m)	119.4	119.4
3''	6.76 – 6.67 (1H, m)	6.72 (1H, m)	114.6	114.6
4''	—	—	145.3	145.3
5''	—	—	146.9	147
6''	6.92 (1H, s)	6.91 (1H, s)	111	110.9
7’'	5.10 (1H, d, J = 2.5 Hz)	4. 79 (1H， dd， J = 5. 0， 3. 2 Hz)	72.1	72.1
8’'	4.23 – 4.16 (1H, m)	4.2 (1H, m)	86.1	86.2
9’'	4.14 – 4.07 (2H, m)	4.10 (2H, m)	59.8	60
1'''	—	—	133.3	133.3
2'''	6.76 – 6.67 (1H, m)	6.72 (1H, m)	119.4	119.4
3'''	6.76 – 6.67 (1H, m)	6.72 (1H, m)	114.6	114.6
4'''	—	—	145.3	145.3
5'''	—	—	146.9	147
6'''	6.92 (1H, s)	6.91 (1H, s)	111	110.9
7’''	5.10 (1H, d, J = 2.5 Hz)	4. 79 (1H， dd， J = 5. 0， 3. 2 Hz)	72.1	72.1
8’''	4.23 – 4.16 (1H, m)	4.2 (1H, m)	86.1	86.2
9’''	4.14 – 4.07 2H, m)	4.10 (2H, m)	59.8	60
(3, 5-OCH3)	3.75 (18H, d, J = 8.4 Hz, 3, 3’, 3’’, 5, 5’, 5’’-OCH3)	3.76 (12H, s)	56	56
(3', 5'-OCH3)			56	56
(3'', 5''-OCH3)		3.73 (6H, s)	55.5	55.5
